# Lysosomes mediate the mitochondrial UPR via mTORC1-dependent ATF4 phosphorylation

**DOI:** 10.1038/s41421-023-00589-1

**Published:** 2023-09-07

**Authors:** Terytty Yang Li, Qi Wang, Arwen W. Gao, Xiaoxu Li, Yu Sun, Adrienne Mottis, Minho Shong, Johan Auwerx

**Affiliations:** 1 State Key Laboratory of Genetic Engineering, Shanghai Key Laboratory of Metabolic Remodeling and Health, Laboratory of Longevity and Metabolic Adaptations, Institute of Metabolism and Integrative Biology, Fudan University, Shanghai, China; 2 Laboratory of Integrative Systems Physiology, Interfaculty Institute of Bioengineering, École Polytechnique Fédérale de Lausanne, Lausanne, Switzerland; 3 Division of Endocrinology and Metabolism, Department of Internal Medicine, Chungnam National University College of Medicine, Daejeon, Korea; 4 Present Address: Laboratory Genetic Metabolic Diseases, Amsterdam Gastroenterology, Endocrinology, and Metabolism, Amsterdam UMC, University of Amsterdam, Amsterdam, the Netherlands

**Keywords:** Stress signalling, Phosphorylation, Mitochondria, Lysosomes, Transcription

## Abstract

Lysosomes are central platforms for not only the degradation of macromolecules but also the integration of multiple signaling pathways. However, whether and how lysosomes mediate the mitochondrial stress response (MSR) remain largely unknown. Here, we demonstrate that lysosomal acidification via the vacuolar H^+^-ATPase (v-ATPase) is essential for the transcriptional activation of the mitochondrial unfolded protein response (UPR^mt^). Mitochondrial stress stimulates v-ATPase-mediated lysosomal activation of the mechanistic target of rapamycin complex 1 (mTORC1), which then directly phosphorylates the MSR transcription factor, activating transcription factor 4 (ATF4). Disruption of mTORC1-dependent ATF4 phosphorylation blocks the UPR^mt^, but not other similar stress responses, such as the UPR^ER^. Finally, ATF4 phosphorylation downstream of the v-ATPase/mTORC1 signaling is indispensable for sustaining mitochondrial redox homeostasis and protecting cells from ROS-associated cell death upon mitochondrial stress. Thus, v-ATPase/mTORC1-mediated ATF4 phosphorylation via lysosomes links mitochondrial stress to UPR^mt^ activation and mitochondrial function resilience.

## Introduction

Long known as the degradative endpoints for intra- and extracellular cargos, lysosomes have emerged as signaling centers that play important roles in nutrient sensing, cell growth, energy metabolism, immune response and aging^1–5^. Accordingly, dysfunction of lysosomes has been associated with a variety of diseases, including lysosomal storage disorders, cancer, diseases of the immune system, and neurodegenerative disorders^6–8^. To maintain energy homeostasis and protein quality control, lysosomes also constantly communicate with other cellular organelles, such as the mitochondria^9,10^. For example, severe mitochondrial dysfunction triggers mitophagy^11^, which results in the degradation of impaired mitochondria by the lysosomes; whereas changes in lysosomal pH or signaling may in turn modulate mitochondrial function and regulate longevity in different organisms^12,13^. However, whether and how the lysosomes mediate the communication from stressed mitochondria to the nucleus are still poorly understood.

The mitochondrial unfolded protein response (UPR^mt^), a branch of the mitochondrial stress response (MSR), is an adaptive transcriptional response that helps to resolve proteostatic toxicity triggered by diverse mitochondrial stresses^14–17^. Although first discovered in mammalian cells^15^, the regulatory mechanisms of UPR^mt^ have been particularly well-studied in the nematode *Caenorhabditis elegans* (*C. elegans*). In *C. elegans*, a panel of transcription factors/co-factors, histone methyltransferases, demethylases, acetyltransferases and deacetylase cooperate with the master UPR^mt^ transcription factor, activated transcription factor-1 (ATFS-1), to mediate the UPR^mt^ upon mitochondrial perturbations^15,18–21^. Nevertheless, how the mitochondrial stress signal is relayed through the cytosol and sensed by these UPR^mt^ regulators is largely unclear. In mammalian cells, mitochondrial stress triggers the integrated stress response (ISR)^22,23^, in which phosphorylation of the eukaryotic translation initiation factor 2α (EIF2α) results in the translation of several transcription factors including activating transcription factor 4 (ATF4), activating transcription factor 5 (ATF5) and C/EBP homologous protein (CHOP) to coordinate a gene expression program considered as the functional equivalent of the UPR^mt,^^14,15,24^. In a parallel study conducted in *C. elegans*, we revealed that increased ATFS-1 translation, mediated by the v-ATPase/TORC1 and lysosomes, contributes to the cytosolic relay of mitochondrial stress to direct UPR^mt^ activation through an EIF2α phosphorylation-independent mechanism^25^. However, whether the roles of lysosomes and v-ATPase/TORC1 in UPR^mt^ regulation are evolutionally conserved remains elusive. Furthermore, how mammalian cells distinguish stress signals from different origins, such as the mitochondrion and ER, to concordantly activate the ATF4-mediated “integrated stress response” is still unknown.

## Results

### Suppression of lysosomal acidification inhibits UPR^mt^ in mammalian cells

**Fig. 1:**
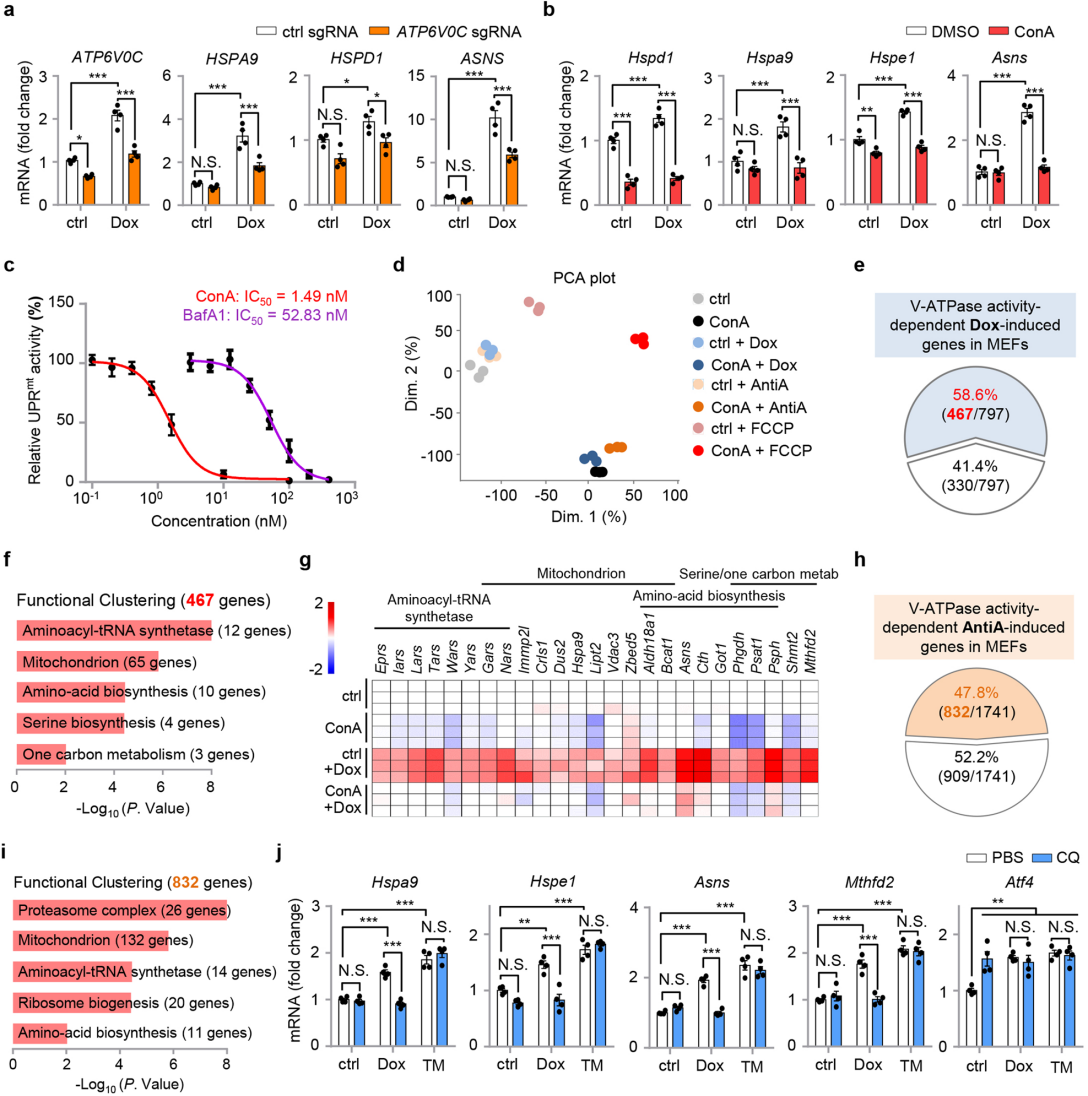
Suppression of lysosomal acidification inhibits UPR^mt^ activation in mammalian cells. **a** qRT-PCR results (*n* = 4 biologically independent samples) of HEK293T cells expressing control (ctrl) or *ATP6V0C* sgRNA, and treated with or without Doxycycline (Dox) (30 μg/mL) for 24 h. **b** qRT-PCR results (*n* = 4 biologically independent samples) of MEFs pretreated with DMSO control or v-ATPase inhibitor Concanamycin A (ConA) (200 nM) for 1 h, and then co-treated with or without Dox (30 μg/mL) for 24 h. **c** The dose-response curve and IC_50_ of ConA (red) and BafA1 (purple) on inhibiting Dox-induced UPR^mt^ activation in MEFs. The relative UPR^mt^ activity was normalized to the mRNA induction level of *Hspa9* (*n* = 4 biologically independent samples) in response to Dox (30 μg/mL, 24 h), and with ConA or BafA1 co-treatment. **d** Principal Component Analysis (PCA) of the RNA-seq profiles of MEFs treated with Dox, Antimycin A (AntiA), FCCP, and/or ConA. **e** Diagram of the UPR^mt^ genes that are dependent (red) on v-ATPase activity for induction upon Dox treatment, according to the RNA-seq dataset. **f** Functional clustering of the 467 v-ATPase activity-dependent genes as indicated in (**e**). **g** Heatmap of the relative expression levels of representative v-ATPase activity-dependent UPR^mt^ genes in MEFs treated with Dox and/or ConA in log_2_ fold change, based on the RNA-seq dataset. See Supplementary Table S1 for detailed gene expression changes. **h** Diagram of the UPR^mt^ genes that are dependent (orange) on v-ATPase activity for induction upon AntiA treatment. **i** Functional clustering of the 832 v-ATPase activity-dependent genes as indicated in (**h**). **j** qRT-PCR results (*n* = 4 biologically independent samples) of MEFs pretreated with PBS control or 50 μM Chloroquine (CQ) for 1 h, and then co-treated with or without Dox (30 μg/mL) or Tunicamycin (TM, 1.5 μg/mL) for 24 h. Error bars denote SEM. Statistical analysis was performed by ANOVA followed by Tukey post hoc test (*P < 0.05; **P < 0.01; ***P < 0.001; N.S. not significant).

The vacuolar H^+^-ATPase (v-ATPase) is a highly conserved large complex proton pump which locates at the lysosomal surface and is essential for the acidification of lysosomes^26,27^. In addition to its role as a proton pump, v-ATPase has also been shown to be crucial for the integration of multiple signaling pathways, including mechanistic target of rapamycin complex 1 (mTORC1)^1,28^, adenosine monophosphate-activated protein kinase (AMPK)^29,30^, as well as Janus kinase 2 (JAK2)-signal transducer and activator of transcription-3 (STAT3) signaling^31^. We first questioned if the role of v-ATPase in UPR^mt^ is functionally conserved in mammalian cells. Doxycycline (Dox)^19,32^, an antibiotic that inhibits mitochondrial ribosome translation, activated the MSR and increased the expression of many UPR^mt^ transcripts (e.g., *HSPA9*, *HSPD1,* and *ASNS*) in human embryonic kidney (HEK) 293T cells (Fig. 1a). This response was suppressed by the knockdown of *ATP6V0C* and *ATP6V0D1* (Fig. 1a and Supplementary Fig. S1a), two core subunits of the v-ATPase complex^26,33^. Similarly, inhibition of v-ATPase activity by two small-molecule inhibitors, Bafilomycin A1 (BafA1) and Concanamycin A (ConA)^34,35^, strongly attenuated Dox-induced expression of typical UPR^mt^ genes in mouse embryonic fibroblasts (MEFs) (Fig. 1b and Supplementary Fig. S1b). Among these approaches to suppress UPR^mt^ activation, ConA treatment in MEFs was the most efficacious (Fig. 1b). Strikingly, the half maximal inhibitory concentration (IC_50_) of ConA on inhibiting Dox-induced UPR^mt^ is below 1.5 nM in MEFs, while that of BafA1 is around 50 nM (Fig. 1c), in line with a more prominent effect of ConA in suppressing v-ATPase activity in vitro^35^.

To systematically evaluate the impact of v-ATPase inhibition on the transcriptional activation of the MSR, we performed RNA sequencing (RNA-seq) on total RNA isolated from MEFs treated with DMSO control or ConA for 24 h, in the absence or presence of three mechanistically different mitochondrial stressors: a mitochondrial translation inhibitor, Dox^19,32^; a mitochondrial complex III inhibitor, Antimycin A (AntiA)^36,37^; and a mitochondrial oxidative phosphorylation uncoupler, carbonyl cyanide *p*-(trifluoromethoxy) phenylhydrazone (FCCP)^38^ (Fig. 1d). Dox upregulated 797 transcripts (adjusted *P* value < 0.05), and 736 (92.3%) of them were also induced by AntiA or FCCP (Supplementary Fig. S1c and Table S1). In contrast, FCCP led to the upregulation of 4182 transcripts, and only 1364 (32.6%) of them were commonly shared with those induced by either Dox or AntiA (Supplementary Fig. S1c). As expected, the 443 genes upregulated in response to all the three mitochondrial stress inducers were enriched for mitochondrial surveillance pathways such as “Aminoacyl-tRNA synthetase”, “Amino-acid biosynthesis” and “Mitochondrion” (Supplementary Fig. S1d), in line with previous studies^19,37,39^. Of note, in addition to “Mitochondrion”, the 2818 transcripts induced only by FCCP were also enriched for other organelles, including “Golgi apparatus”, “Endosome”, and “Endoplasmic reticulum (ER)” (Supplementary Fig. S1e), reminiscent of other metabolic impacts of FCCP or FCCP-like protonophore uncouplers through mechanisms irrelevant of mitochondrial membrane potential disruption^40,41^. Consistently, in the Principal Component Analysis (PCA) plot of the RNA-seq dataset (Fig. 1d), the FCCP-induced cellular response in overall gene expression is much more pronounced as compared to Dox or AntiA treatment, in line with a much larger differentially expressed gene (DEG) list upon FCCP treatment than that of Dox or AntiA (Supplementary Fig. S1c).

As expected, a more restricted number of transcripts was altered upon Dox stimulation in the presence of ConA (Supplementary Fig. S1f, g). Importantly, among the 797 Dox-induced transcripts, a majority (58.6%, 467 transcripts) of them was abrogated by ConA (Fig. 1e). These 467 genes, hereby defined as the “v-ATPase activity-dependent Dox-induced genes”, were enriched for MSR-related pathways including “Aminoacyl-tRNA synthetase” (e.g., *Tars* and *Nars*), “Mitochondrion” (e.g., *Hspa9*, a classical UPR^mt^ reporter gene), “Amino-acid biosynthesis” (e.g., *Asns*), “Serine biosynthesis” (including *Phgdh*, *Psat1,* and *Psph*, encode three rate-limiting genes for de novo serine biosynthesis) and “One carbon metabolism” (e.g., *Shmt2* and *Mthfd2*) (Fig. 1f, g). Likewise, around half (47.8%) of the AntiA-induced transcripts relied on v-ATPase activity for induction (Fig. 1h). Despite that only 30.4% of the FCCP-induced transcripts were abrogated by ConA (Supplementary Fig. S1h), the 832 AntiA-induced and 1271 FCCP-induced transcripts that were dependent on v-ATPase activity were both enriched for mitochondrion-related pathways (Fig. 1i and Supplementary Fig. S1i). In contrast, the other 2911 FCCP-induced transcripts independent of v-ATPase activity were highly enriched for other cellular organelles such as “Endoplasmic reticulum”, “Golgi apparatus”, “Lysosome” and “Endosome” (Supplementary Fig. S1j), suggesting that the v-ATPase inhibitor ConA probably attenuated the adaptive response specifically related to mitochondria, but not other cellular organelles such as the ER.

Consistently, ConA inhibited the induction of UPR^mt^ genes, including *Hspa9*, *Asns*, *Psph,* and *Mthfd2*, upon exposure to mitochondrial stress inducers, AntiA and Oligomycin (Olig) (Supplementary Fig. S1k). Despite that the ER UPR (UPR^ER^) inducer Tunicamycin (TM) also upregulated these MSR transcripts, as reported previously^42^, their induction upon TM treatment was surprisingly not affected by ConA (Supplementary Fig. S1k). Moreover, the TM-induced robust upregulation of ER chaperone *Grp78/Bip*^43^, a key event of the UPR^ER^ ^44,45^, was also not affected by ConA (Supplementary Fig. S1k). As an alternative approach to inhibit lysosome acidification and mimic v-ATPase loss-of-function, disruption of lysosomal pH gradient by chloroquine (CQ)^46^, also abrogated Dox-induced UPR^mt^ activation, but not the TM-induced stress response (Fig. 1j). Suppression of lysosome acidification also disrupted iron metabolism and induced the expression of multiple iron homeostasis-related genes (Supplementary Fig. S1l, m), in line with previous reports^47,48^. Interestingly, different from the effects of ConA and CQ, the mTOR inhibitors Rapamycin and Torin1^49^, robustly attenuated the induction of typical UPR^mt^ and UPR^ER^ genes in response to Dox or TM treatment^42^, and inhibited the expression of key transcription factors *Atf4*, *Atf5* and *Chop* (Supplementary Fig. S1n). Together, these results suggest that disruption of lysosomal acidification by inhibiting v-ATPase activity or CQ suppresses the UPR^mt^, but not other similar stress responses such as the UPR^ER^, while direct mTORC1 inhibition abrogates the transcriptional responses induced by both mitochondrial and ER stress inducers.

### Lysosomal inhibition increases ATF4 accumulation but also limits ATF4 binding to the promoters of UPR^mt^ genes

**Fig. 2:**
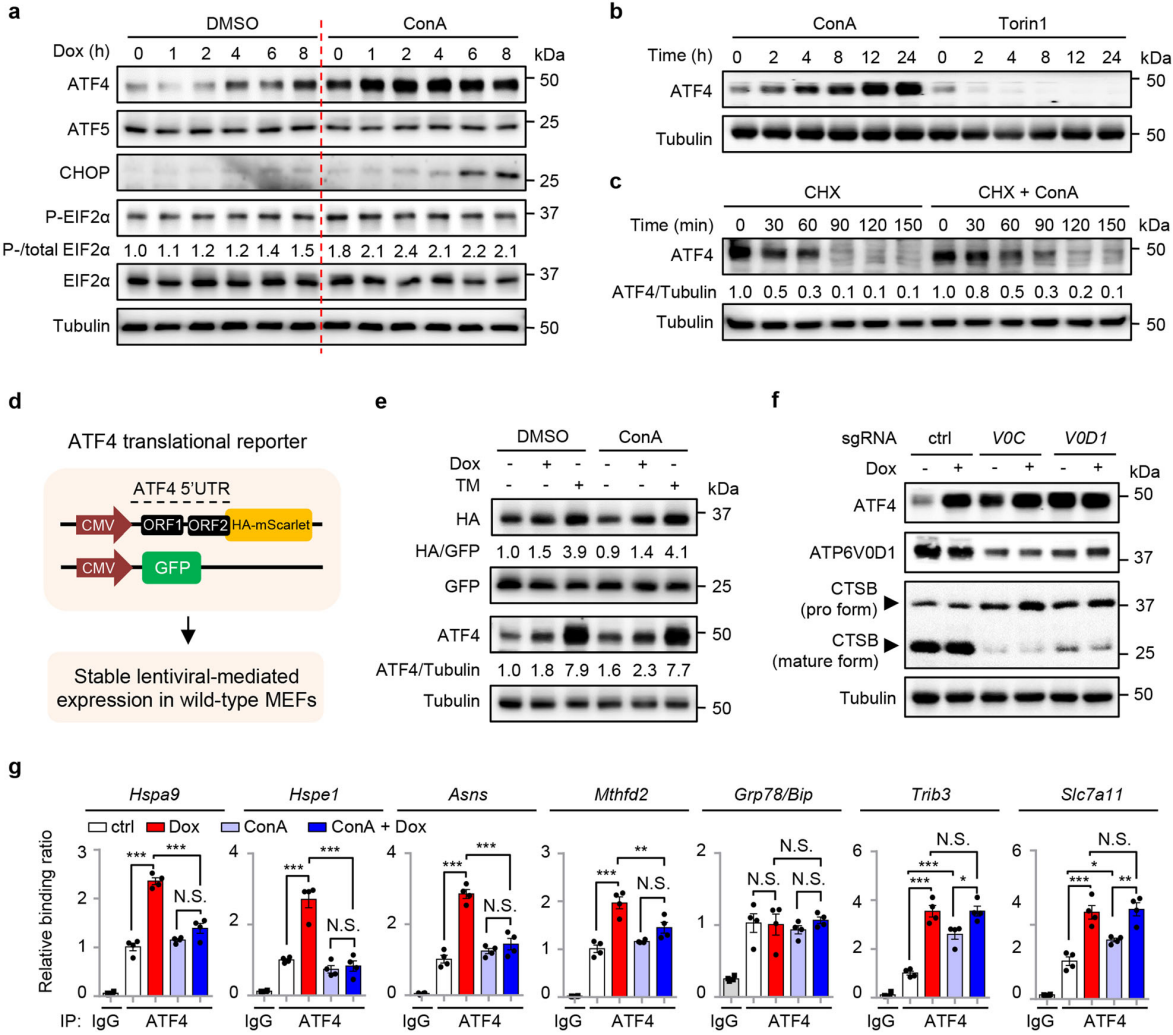
Lysosomal inhibition increases ATF4 accumulation but also limits ATF4 binding to the promoters of UPR^mt^ genes. **a** Western blot analysis showing time-dependent changes of proteins in MEFs pretreated with DMSO control or ConA (200 nM) for 1 h, and then co-treated with Dox (30 μg/mL) for 0–8 h. All ConA-treated conditions were thus treated with ConA for a total time of 9 h. **b** Western blot analysis of MEFs treated with ConA (200 nM) or Torin1 (250 nM) for 0–24 h. **c** Western blot analysis of MEFs treated with cycloheximide (CHX), in the absence or presence of ConA (200 nM) for 0–150 min. **d** Schematic diagram of the ATF4 translational reporter, comprising the upstream open reading frames (uORF1 and uORF2) of the ATF4 5' untranslated region (5′ UTR) followed by HA-mScarlet tag replacing the ATF4 coding sequence, built on a lentiviral expression vector. The GFP control is directly driven by the cytomegalovirus (CMV) promoter. **e** Western blot analysis of MEFs stably expressing the ATF4 translational reporter and GFP control treated with or without Dox (30 μg/mL) or Tunicamycin (TM, 1.5 μg/mL) for 3 h, in the presence of DMSO control or ConA (200 nM). **f** Western blot analysis of HEK293T cells expressing control (ctrl), *ATP6V0C* (*V0C*) or *ATP6V0D1* (*V0D1*) sgRNA, and treated with or without Dox (30 μg/mL) for 24 h. The pro and mature forms of Cathepsin B (CTSB) were as indicated. **g** ATF4 ChIP-qPCR analysis (*n* = 4 biologically independent samples) of the promoters of ATF4-targeted genes in MEFs pretreated with DMSO or ConA (200 nM) for 1 h, and then co-treated with or without Dox (30 μg/mL) for 3 h. Error bars denote SEM. Statistical analysis was performed by ANOVA followed by Tukey post hoc test (*P < 0.05; **P < 0.01; ***P < 0.001; N.S. not significant).

To investigate the mechanism underlying how lysosomes and v-ATPase regulate the UPR^mt^, we first checked the expression levels of the putative UPR^mt^ transcription factors (i.e., ATF4, ATF5, and CHOP) upon ConA treatment, with or without mitochondrial stress. Surprisingly, their mRNA levels were upregulated even in cells with only ConA treatment (Supplementary Fig. S2a, b); the expression of multiple classical ATF4 targets, including *Chac1*, *Herpud1*, *Trib3,* and *Slc7a11*, that are induced in response to ER stress^39,42^, were increased under this condition as well (Supplementary Fig. S2b). Similar patterns were also found for the mitophagy/autophagy transcripts (e.g., *Sqstm1*, *Binp3l*, *Pink1*) (Supplementary Fig. S2b). At the protein level, Dox mildly increased EIF2α phosphorylation and ATF4 expression^39^ (Fig. 2a and Supplementary Fig. S2c). Interestingly, a higher level of ATF4 protein was detected in ConA/Dox co-treated MEFs at all time points, as compared to Dox-only conditions (Fig. 2a). Meanwhile, the expression of ATF5 and CHOP was either not affected or only slightly induced by Dox or ConA (Fig. 2a). Moreover, ConA alone increased ATF4 protein expression in a time-dependent manner (Fig. 2b). In contrast, complete inhibition of mTORC1 activity by Torin1 led to depletion of ATF4 protein (Fig. 2b), in line with its mRNA changes and previous studies^50,51^ (Supplementary Fig. S1n).

To understand how ConA leads to ATF4 protein accumulation, we then measured the protein stability of ATF4 when protein synthesis was blocked by a translation inhibitor, cycloheximide (CHX). The half-life of ATF4 is very short at basal state^50,52^, and was almost doubled (from 30 to 60 min) in the presence of ConA (Fig. 2c and Supplementary Fig. S2d). Next, by using an ATF4 translation reporter^53^, which expresses an HA-tagged reporter protein under the strict control of ATF4 5’ UTR (Fig. 2d), we found that ConA in general did not affect the translation of ATF4 (as determined by comparing the expression of the ATF4 5’ UTR-driven HA-tagged reporter protein and the normal CMV5 promoter-driven GFP protein), in response to either Dox or TM treatment for 3 h (Fig. 2e and Supplementary Fig. S2e). Meanwhile, the endogenous expression of ATF4 protein increased by 60% upon only ConA exposure (Fig. 2e and Supplementary Fig. S2f). These results suggest that a basal mTORC1 activity still exists for normal ATF4 translation even with v-ATPase inhibition, and ConA increases ATF4 accumulation through a translation-independent mechanism.

Increased expression of ATF4 was also found in cells with *ATP6V0C* or *ATP6V0D1* knockdown, or CQ treatment (Fig. 2f and Supplementary Fig. S2g). As expected, disrupted lysosomal pH and Cathepsin B (CTSB) maturation, which requires lysosomal activity-dependent proteolytic removal of the pro-domain of cathespins^54^, were detected in cells after the treatment of different lysosomal acidification inhibitors or after *ATP6V0C* or *ATP6V0D1* knockdown (Supplementary Fig. S2h–j). To elucidate how the accumulated ATF4 protein in ConA-treated MEFs failed to activate the UPR^mt^ genes upon mitochondrial stress (Fig. 1g and Supplementary Table S1), we pulled down the endogenous ATF4 in MEFs with or without ConA and/or Dox treatment, and performed a chromatin immunoprecipitation coupled with quantitative PCR (ChIP-qPCR). Dox strongly promoted the enrichment of ATF4 at the loci of multiple UPR^mt^ genes (e.g., *Hspa9*, *Hspe1*, *Asns*, *Mthfd2*), which was almost completely blocked by ConA (Fig. 2g). In contrast, enrichment of ATF4 at the loci of UPR^ER^-related genes was either unchanged (e.g., *Grp78/Bip*) or even increased (e.g., *Trib3*) upon ConA (Fig. 2g), in line with their changes at mRNA level (Supplementary Fig. S2b). Thus, inhibition of lysosomal acidification increases ATF4 accumulation but also limits ATF4 binding to the promoters of UPR^mt^ genes during mitochondrial stress.

Of note, increased level of ATF4 upon ConA treatment or v-ATPase inhibition is most likely the consequence of reduced lysosomal activity-dependent protein degradation of ATF4, for the following reasons: (i), the half-life of ATF4 almost doubled from 30 min to 60 min upon ConA treatment (Fig. 2c), suggesting that the degradation process of ATF4 is strongly attenuated by ConA; (ii), by taking advantage of the ATF4 translational reporter^53^, which expresses an HA-tagged reporter protein mScarlet under the control of ATF4 5’ UTR (Fig. 2d), we found that 3 h of ConA treatment did not affect the translation of this ATF4 reporter (as shown by the ratio of HA/GFP), while the endogenous level of ATF4 was increased by ~60% compared with DMSO control (Fig. 2e); (iii), direct inhibition of lysosomal activity with CQ also leads to ATF4 protein accumulation in a dose-dependent manner (Supplementary Fig. S2g). Together, these results strongly support a model that ConA increases ATF4 accumulation by attenuating its lysosomal activity-dependent protein degradation, rather than by affecting its translation.

### Mitochondrial stress induces a lysosomal activity-dependent mTORC1 activation at the lysosomal surface

**Fig. 3:**
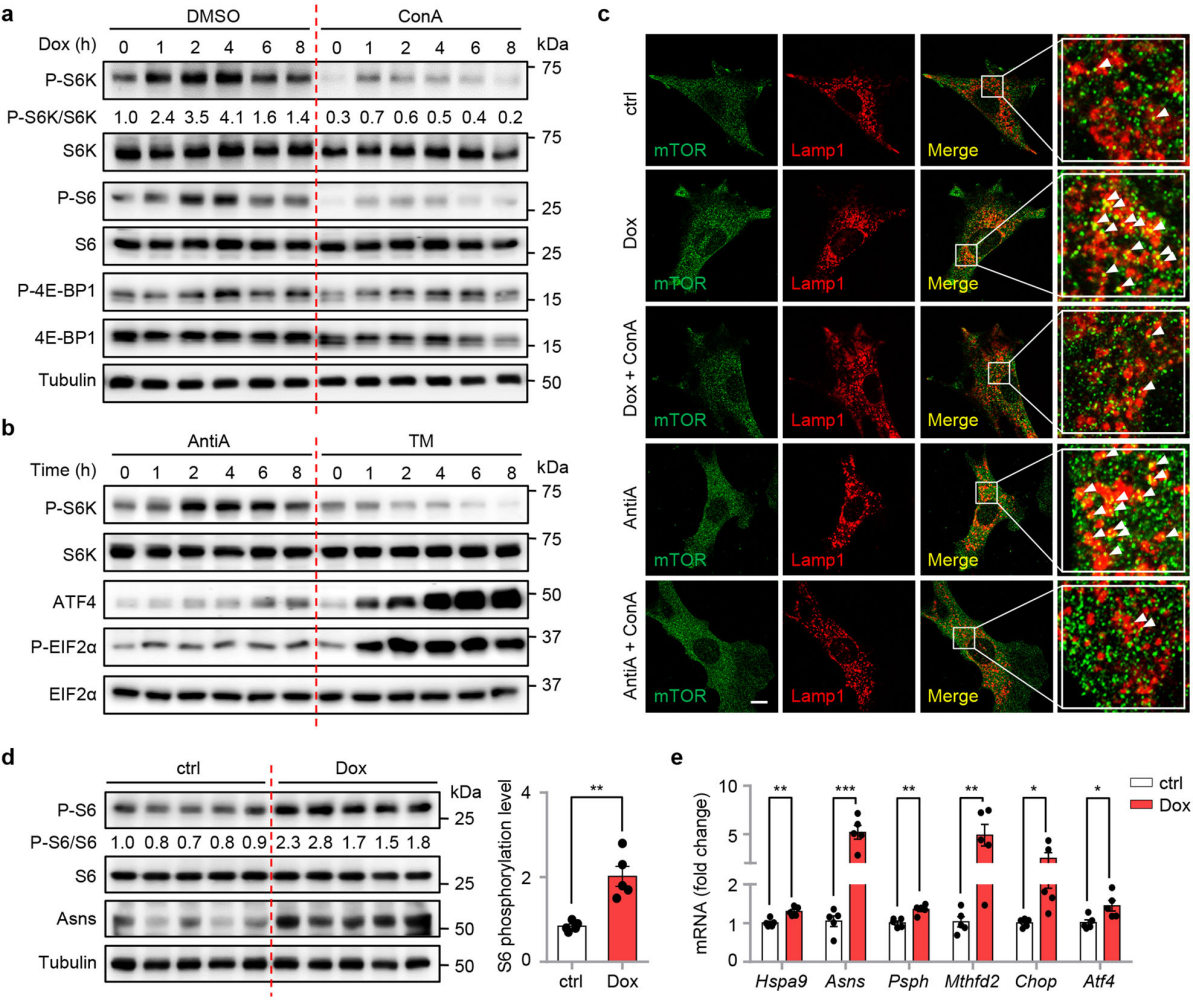
Mitochondrial stress induces v-ATPase-dependent mTORC1 activation at the lysosomal surface. **a** Western blot analysis showing time-dependent changes of proteins in MEFs pretreated with DMSO or ConA (200 nM) for 1 h, and then co-treated with or without Dox (30 μg/mL) for 0–8 h. **b** Western blot analysis showing time-dependent changes of proteins in MEFs treated with Antimycin A (AntiA, 2 μM) or Tunicamycin (TM, 1.5 μg/mL) for 0–8 h. **c** ConA inhibits mitochondrial stress-induced lysosomal localization of mTOR in MEFs. MEFs were pretreated with DMSO control or ConA (200 nM) for 1 h, and then co-treated with or without Dox (30 μg/mL) for 3 h, cells were then fixed and co-stained with mTOR (green) and lysosome marker Lamp1 (red) antibodies. The arrows indicate the mTOR-lysosome co-localized puncta. Scale bar, 10 μm. **d**, **e** Western blot analysis (**d**) and qRT-PCR results (*n* = 5 mice for each group) (**e**) of kidney samples from 9–10-week-old male C57BL/6J mice treated with vehicle control (ctrl) or Dox (50 mg/kg) for 24 h. Error bars denote SEM. Statistical analysis was performed by two-tailed unpaired Student’s *t* test (*P < 0.05; **P < 0.01; ***P < 0.001; N.S. not significant).

Next, we checked whether mTORC1 signaling is activated upon mitochondrial stress, as revealed in our recent studies in human thyroid cancer cells and in *C. elegans*^25,55^. mTORC1 activity (as reflected by the phosphorylation of S6K, S6, and 4E-BP1) increased and peaked at 2–4 h of Dox treatment in MEFs, which was attenuated by the v-ATPase inhibitor ConA (Fig. 3a and Supplementary Fig. S3a). A similar time-dependent activation pattern of mTORC1 was also observed in MEFs treated with AntiA (Fig. 3b), or with Olig treatment as reported eleswhere^56^. In contrast, the ER stress inducer TM gradually decreases S6K phosphorylation in MEFs (Fig. 3b), consistent with previous studies^42,57^. In line with the model that a mitochondrion-endosome-lysosome route that shuttles cargo from mitochondria to lysosomes is activated upon oxidative stress^58^, the mitochondrial dye MitoTracker^59^ strongly co-localized with early endosome vesicles (Rab5^+^), and partially with late endosome vesicles (Rab7^+^), but not with mature lysosomes (Lamp1^+^), 3 h after Dox treatment (Supplementary Fig. S3b–d). This phenomenon is likely caused by the breakdown of mitochondria in the more acidic late endosomes or lysosomes, which in turn resulted in the loss of the mitochondrial signal^58,60^. mTORC1 activation requires its dynamic recruitment to the lysosomal surface^28^. As expected, both Dox and AntiA promoted lysosomal, but not early or late endosomal, localization of mTORC1, which was furthermore suppressed by ConA (Fig. 3c and Supplementary Fig. S3e, f). Finally, increased mTORC1 activity, as reflected by the S6 phosphorylation, was also detected in vivo in the kidneys of wild-type C57BL/6J mice upon Dox administration (Fig. 3d), in accordance with the upregulation of UPR^mt^ genes (Fig. 3d, e). These results suggest that mitochondrial stress induces a time-dependent activation of mTORC1 signaling, which furthermore relies on the intact function of v-ATPase and the lysosomes.

### ATF4 is a direct phosphorylation target of mTOR in response to mitochondrial stress

**Fig. 4:**
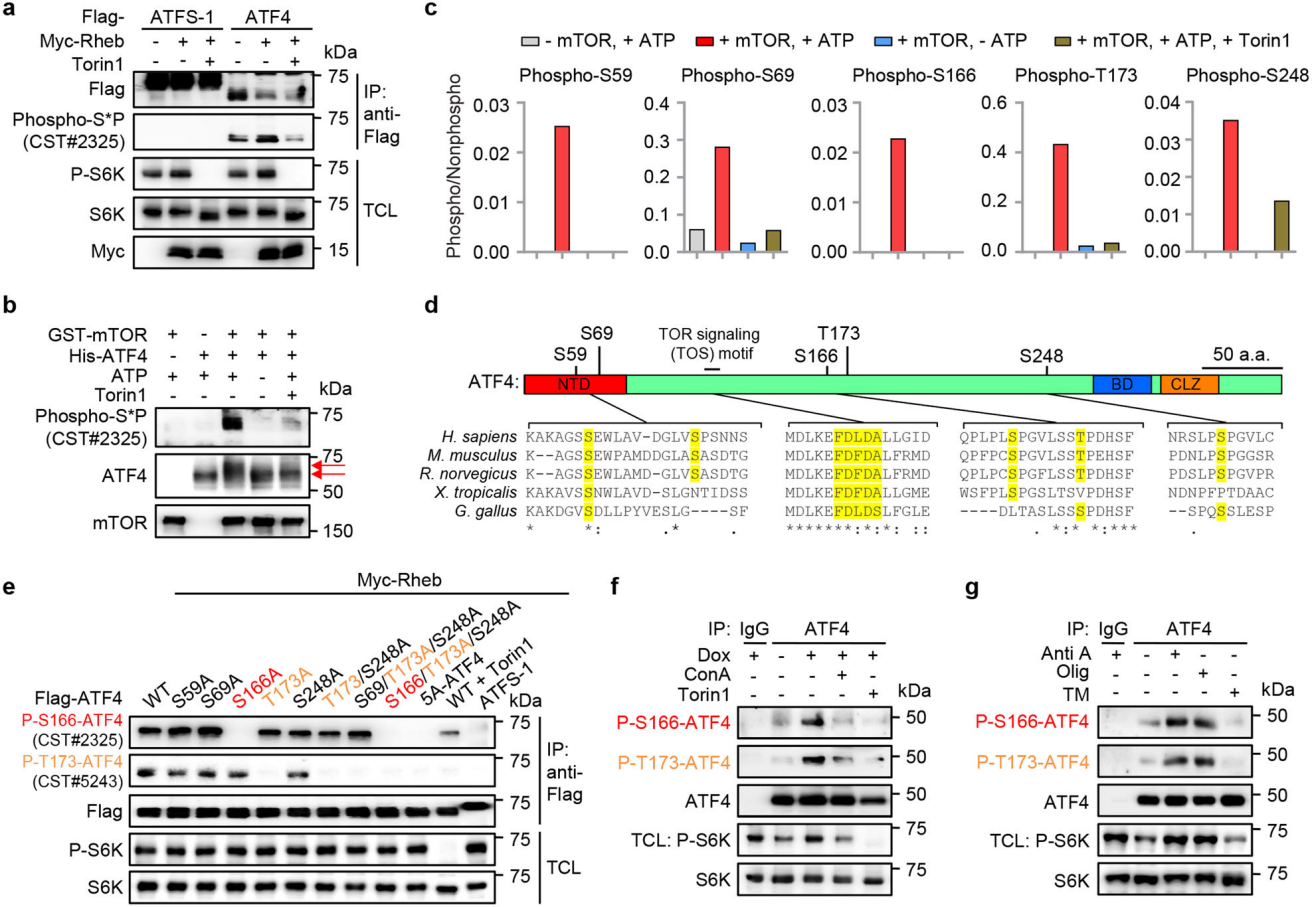
ATF4 is a direct phosphorylation target of mTOR in response to mitochondrial stress. **a** ATF4 phosphorylation recognized by a context-dependent (S*P) phosphorylation-specific antibody is increased by Rheb co-expression and inhibited by Torin1. HEK293T cells transfected with the indicated plasmids were immunoprecipitated (IP) with anti-Flag antibody and analyzed by western blot analysis. When applicable, Torin1 (250 nM) were added 2 h before harvest. TCL total cell lysate. **b** mTOR directly phosphorylates ATF4 in vitro. In vitro kinase assay was performed with recombinant GST-tagged human mTOR purified from baculovirus-infected insect cells and recombinant His-tagged human ATF4 with or without Torin1 (250 nM). Arrows indicate the mobility shifts likely separating the hyperphosphorylated and nonphosphorylated ATF4. **c** The ratios of the phosphorylated and nonphosphorylated peptides containing the phosphorylation sites of ATF4 from a kinase assay performed similar to (**b**), as determined by mass spectrometry. **d** The identified mTOR-targeted phosphorylation sites on ATF4 with the vertebrate orthologs aligned below, with numbering according to the amino-acid sequence of human ATF4 protein. NTD N-terminal domain, BD Basic domain, CLZ C-leucine zipper. The highly conserved putative TOR signaling (TOS) motif was also highlighted. **e** Validation of the two commercially available antibodies that specifically recognize ATF4 phosphorylation at Ser^166^ and Thr^173^, respectively. HEK293T cells transfected with the indicated plasmids were immunoprecipitated with anti-Flag antibody and analyzed by western blot assay. Torin1 (250 nM) was added 2 h before harvest. **f** Increased ATF4 Ser^166^ and Thr^173^ phosphorylation upon Dox treatment, which was inhibited by ConA and Torin1. Wild-type MEFs were pretreated with DMSO, ConA (200 nM) or Torin1 (250 nM) for 1 h, and then co-treated with or without Dox (30 μg/mL) for 2 h, immunoprecipitated with anti-ATF4 antibody and analyzed by western blot assay. **g** Increased ATF4 phosphorylation upon mitochondrial, but not ER stress inducers. Wild-type MEFs were with treated with Antimycin A (AntiA, 2 μM), Oligomycin (Olig, 2 μM), or Tunicamycin (TM, 1.5 μg/mL) for 2 h, immunoprecipitated with anti-ATF4 antibody and analyzed by western blot assay. A similar amount of immunoprecipitated ATF4 protein was loaded for different conditions to compare phosphorylation changes in (**f**, **g**).

The fact that the accumulated ATF4 in ConA-treated MEFs failed to activate UPR^mt^ hints to the existence of certain post-translational modifications of ATF4, which are regulated in a v-ATPase/mTORC1-dependent fashion. In light of the kinase nature of mTOR, we questioned whether ATF4 is a direct phosphorylation target of mTORC1. Co-expression of ATF4 with the mTORC1 upstream activator Rheb increased the phosphorylation signal that was detected by a context-dependent (S*P) phosphorylation-specific antibody, which was inhibited by Torin1 (Fig. 4a). In contrast, no apparent phosphorylation signal was found when ATFS-1, the master transcription factor of UPR^mt^ in *C. elegans*^18^, was co-expressed with or without Rheb (Fig. 4a). Importantly, mTORC1-dependent phosphorylation of ATF4 was also detected in an in vitro kinase assay using either the mTORC1 immunoprecipitated from HEK293T cells or a recombinant kinase active mTOR protein purified from insect cells (Fig. 4b and Supplementary Fig. S4a). Mass spectrometric analysis revealed the existence of 5 serine/threonine (S/T) sites on ATF4 that can be phosphorylated by mTOR, and are sensitive to Torin1 treatment (Fig. 4c and Supplementary Fig. S4b). These sites are in general well-conserved across vertebrate species (Fig. 4d). It has been reported that mTORC1 substrates including S6K and 4E-BP1 harbor a canonical five amino-acid TOR signaling (TOS) motif that is crucial for their regulation by mTORC1^61,62^. We discovered that a highly conserved TOS motif (-FDLDA-) also exists in the N-terminal of ATF4 protein (Fig. 4d), supporting that ATF4 is a bona fide evolutionally conserved phosphorylation target of mTOR. Separated or combined mutation of the five phosphorylation candidate sites to alanine revealed that ATF4 phosphorylation at Ser^166^ was specifically recognized by the context-dependent (S*P) phosphorylation antibody (referred hereafter as P-S166-ATF4 antibody), while P-T173-ATF4 was revealed by another context-dependent (ST*P) antibody (Fig. 4e). Moreover, Dox increased the endogenous P-S166-ATF4 and P-T173-ATF4 levels, which were furthermore abrogated by both ConA and Torin1 (Fig. 4f). Finally, increased phosphorylation of ATF4 at S166 and T173 was also detected upon exposure to the mitochondrial stress inducers such as AntiA and Olig (Fig. 4g), while ER stress inducer TM suppressed ATF4 phosphorylation, consistent with the changes of S6K phosphorylation (Fig. 4g). Thus, ATF4 is a direct phosphorylation target of mTORC1 downstream of multiple UPR^mt^ activators, but not the UPR^ER^ inducer TM.

### An essential role of ATF4 and its phosphorylation by mTORC1 in UPR^mt^ activation

**Fig. 5:**
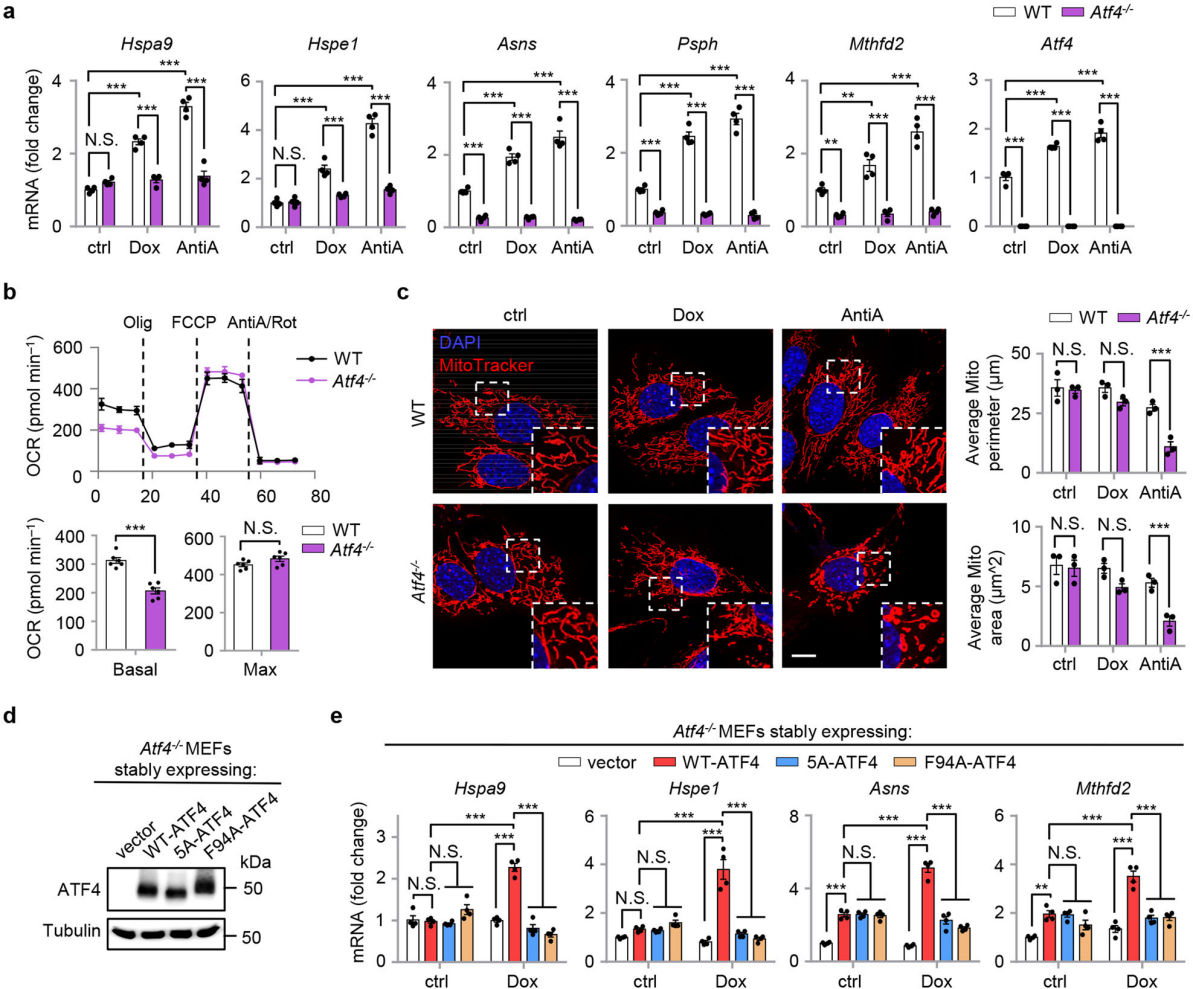
An essential role of ATF4 and its phosphorylation by mTORC1 in UPR^mt^ activation. **a** qRT-PCR results (*n* = 4 biologically independent samples) of wild-type (WT) and *Atf4*^*−/−*^ MEFs treated with or without Dox (30 μg/mL) or Antimycin A (AntiA, 2 μM) for 24 h. **b** The OCR of WT or *Atf4*^*−/−*^ MEFs at basal or after sequential addition of Oligomycin (Olig), FCCP and AntiA/Rotenone. The basal and maximum OCR was statistically analyzed (*n* = 6 biologically independent samples). **c** *Atf4* knockout leads to disrupted mitochondrial network upon mitochondrial stress. MitoTracker staining of WT or *Atf4*^*−/−*^ MEFs treated with or without Dox (30 μg/mL) or AntiA (2 μM) for 24 h. The average mitochondrial network perimeter and area were analyzed by ImageJ with a Mito-Morphology macro (*n* = 3 independent experiments). Scale bar, 10 μm. **d** Western blot analysis of *Atf4*^*−/−*^ MEFs stably expressing empty vector (vector), the wild-type ATF4 (WT-ATF4), the phospho-defective mutant (5A-ATF4), and an ATF4 mutant carrying a point mutation of the bulky phenylalanine residue 94 in the TOS motif to alanine (F94A-ATF4). **e** qRT-PCR results (*n* = 4 biologically independent samples) of *Atf4*^*−/−*^ MEFs stably expressing vector, wild-type, 5A or F94A forms of ATF4, treated with or without Dox (30 μg/mL) for 24 h. Error bars denote SEM. Statistical analysis was performed by two-tailed unpaired Student’s *t* test in (**b**), or by ANOVA followed by Tukey post hoc test in (**a**, **c**, **e**) (**P < 0.01; ***P < 0.001; N.S. not significant).

Characterization of *Atf4*^*−/−*^ MEFs revealed an essential role of ATF4 in the induction of typical UPR^mt^ genes in response to Dox or AntiA treatment (Fig. 5a). Furthermore, certain UPR^mt^ genes (e.g., *Asns*, *Psph*, *Mthfd2*) rely on ATF4 for basal expression (Fig. 5a), in line with a previous study^63^. While the basal oxygen consumption rate (OCR) was remarkably decreased in *Atf4*^*−/−*^ MEFs as compared to that in WT cells, the maximum OCR after acute FCCP treatment was not affected (Fig. 5b), supporting a global metabolic reprogramming upon *ATF4* loss-of-function^39,63^. Whereas in the basal state, the mitochondrial network in *Atf4*^*−/−*^ MEFs is similar to that in WT cells (Fig. 5c), the mitochondrial network tended to be more disrupted in *Atf4*^*−/−*^ MEFs upon Dox administration, an effect that was even more pronounced after AntiA exposure (Fig. 5c). The different impacts of Dox and AntiA on the mitochondrial network are also in line with a more potent effect of AntiA in regulating gene expression and UPR^mt^ activation (Fig. 5a and Supplementary Fig. S1c).

We then reconstituted the *Atf4*^*−/−*^ MEFs with either an empty vector control (vector), wild-type ATF4 (WT-ATF4), the ATF4 phosphorylation defective mutant (5A-ATF4, with all five serine/threonine phosphorylation sites mutated to alanine) or an ATF4 mutant carrying a point mutation of the bulky phenylalanine residue 94 (numbering for human ATF4) in the TOS motif to alanine (F94A-ATF4) (Fig. 5d). The phenylalanine to alanine mutation in the TOS motif of canonical mTOR substrates typically disrupts the functional impact of mTORC1 on its substrates, including on S6K and 4E-BP1^61,62^. As expected, in contrast to WT-ATF4, both 5A-ATF4 and F94A-ATF4 were unable to activate the UPR^mt^ in *Atf4*^*−/−*^ MEFs upon Dox treatment (Fig. 5e). Moreover, these ATF4 mutants failed to increase their binding to the promoters of UPR^mt^ genes in response to Dox (Supplementary Fig. S5a), in line with the results with ConA treatment (Fig. 2h). Furthermore, the TM-induced expression of the representative UPR^mt^/UPR^ER^-shared genes (i.e., *Hspa9*, *Hspe1*, *Asns,* and *Mthfd2*) or the UPR^ER^-specific target *Grp78/Bip* was not affected in 5A-ATF4 or F94A-ATF4-rescued *Atf4*^*−/−*^ MEFs, relative to WT-ATF4-rescued MEFs (Supplementary Fig. S5b). Meanwhile, the expression of *Hspa9* and *Hspe1* was still significantly increased upon TM treatment, even in *Atf4*^*−/−*^ MEFs rescued with the empty control vector (Supplementary Fig. S5b), confirming the involvement of other transcription factors (e.g., ATF6 and XBP1s) in ER stress response^44,45,63^. Finally, *Atf4*^*−/−*^ MEFs reconstituted with a phosphomimetic ATF4 mutant (5D-ATF4, with all the five serine/threonine sites mutated to aspartic acid) displayed constitutively activated UPR^mt^ even under basal condition, and Dox did not further activate the UPR^mt^ to a higher extent (Supplementary Fig. S5c, d). Thus, both ATF4 and its phosphorylation by mTORC1 are essential for mitochondrial stress-induced UPR^mt^ activation.

### mTORC1-mediated ATF4 phosphorylation sustains mitochondrial homeostasis and protects cells from ROS-associated cell death upon mitochondrial stress

**Fig. 6:**
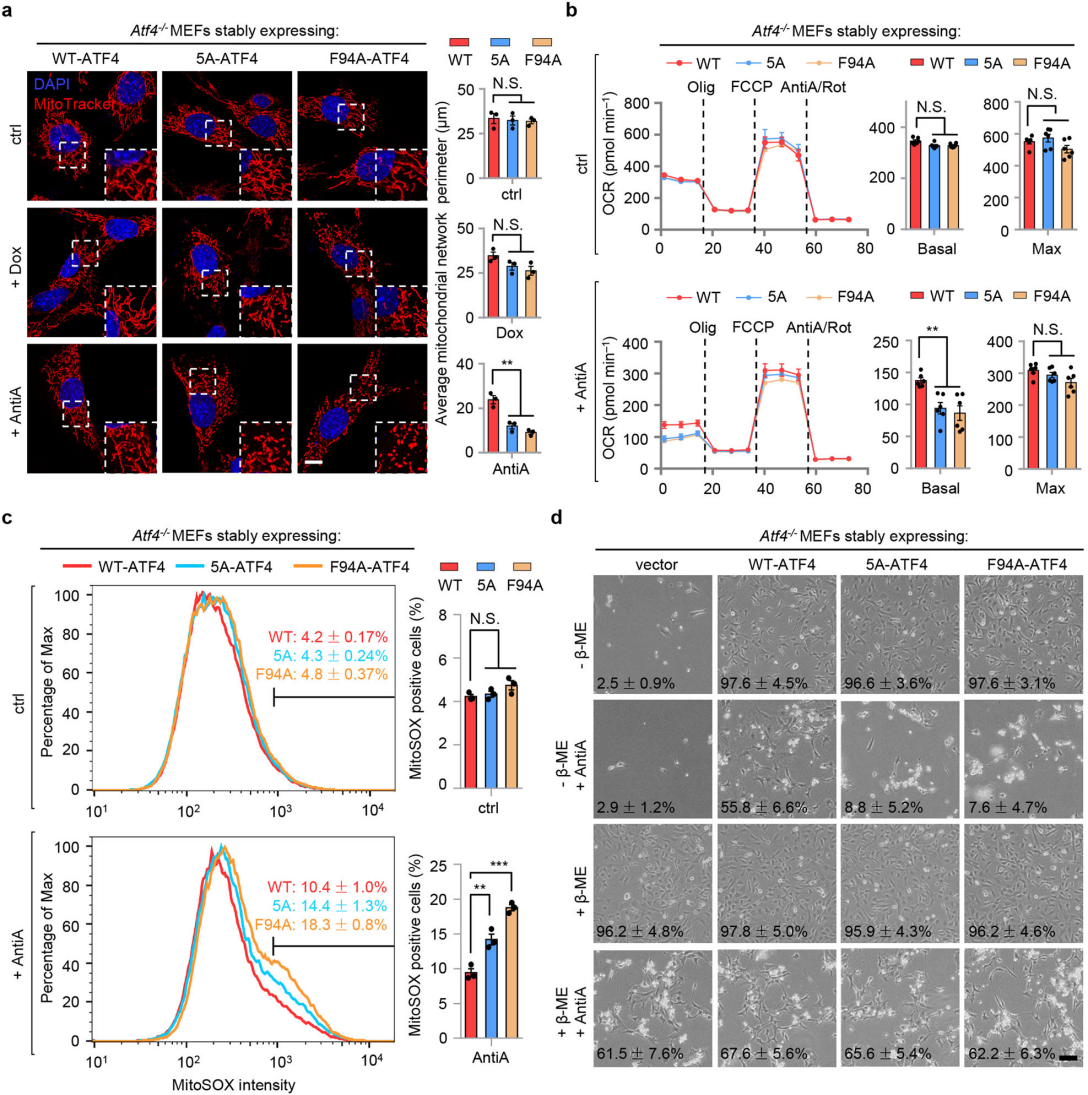
mTORC1-mediated ATF4 phosphorylation sustains mitochondrial homeostasis and protects cells from ROS-associated cell death upon mitochondrial stress. **a** MitoTracker staining of *Atf4*^*−/−*^ MEFs stably expressing wild-type (WT), 5A or F94A forms of ATF4, with or without Dox (30 μg/mL) or AntiA (2 μM) treatment for 24 h. The average mitochondrial network perimeter and area were analyzed by ImageJ with a Mito-Morphology macro (*n* = 3 independent experiments). Scale bar, 10 μm. **b** The OCR of *Atf4*^*−/−*^ MEFs stably expressing WT, 5A or F94A forms of ATF4, after DMSO control (ctrl) or AntiA (2 μM) treatment for 24 h. The basal and maximum OCR was analyzed (*n* = 6 biologically independent samples). **c** Flow cytometry analysis of the mitochondrial superoxide (MitoSOX) intensity of *Atf4*^*−/−*^ MEFs stably expressing WT, 5A or F94A forms of ATF4, after DMSO control or AntiA (2 μM) exposure for 48 h. The percentages of MitoSOX-positive cells were analyzed (*n* = 3 independent experiments). **d** Representative bright field photographs of *Atf4*^*−/−*^ MEFs stably expressing empty vector, WT, 5A or F94A forms of ATF4, grown with or without the antioxidant β-mercaptoethanol (β-ME) or AntiA (2 μM) for 96 h. Mean percentages (±SEM) of the survival ratio of cells are indicated (*n* = 3 independent experiments). Error bars denote SEM. Statistical analysis was performed by ANOVA followed by Tukey post hoc test (**P < 0.01; ***P < 0.001; N.S. not significant).

We then assessed the role of mTORC1-mediated ATF4 phosphorylation in mitochondrial homeostasis and function. In line with the findings in *Atf4*^*−/−*^ MEFs (Fig. 5c), healthy and well-connected mitochondrial network still exists in *Atf4*^*−/−*^ MEFs reconstituted with WT-ATF4, the 5A-ATF4 or F94A-ATF4 at basal state (Fig. 6a). A trend towards disrupted mitochondrial network upon Dox treatment was seen in 5A-ATF4- or F94A-ATF4-rescued *Atf4*^*−/−*^ MEFs, but not in those rescued with WT-ATF4; this tendency became more pronounced after the cells were challenged with AntiA (Fig. 6a). Consistently, reduced OCR was found in 5A-ATF4 or F94A-ATF4-rescued *Atf4*^*−/−*^ MEFs, compared to that in MEFs rescued with WT-ATF4 after AntiA exposure (Fig. 6b). By tracking the mitochondrial reactive oxygen species (ROS) level with MitoSOX^64^, remarkably higher percentages of MitoSOX-positive cells were detected in *Atf4*^*−/−*^ MEFs expressing 5A-ATF4 and F94A-ATF4 after AntiA treatment, compared to that in cells expressing WT-ATF4 (Fig. 6c and Supplementary Fig. S6). Finally, the *Atf4*^*−/−*^ MEFs reconstituted with 5A-ATF4 or F94A-ATF4 were more prone to AntiA-induced cell death, which was rescued with the supplement of the antioxidant, β-mercaptoethanol (β-ME) (Fig. 6d). Of note, in contrast to the *Atf4*^*−/−*^ MEFs which require the addition of both the non-essential amino acids and certain antioxidants (e.g., β-ME) in the culture media to maintain their survival^63^, *Atf4*^*−/−*^ MEFs rescued with 5A-ATF4 or F94A-ATF4 grow as well as those rescued with WT-ATF4 even without these supplements at unstressed condition (Fig. 6d). We have also noticed that *Atf4*^*−/−*^ MEFs rescued with F94A-ATF4 demonstrated more severe defects in mitochondrial function upon mitochondrial stress (Fig. 6a–c), as compared to that in cells rescued with 5A-ATF4, suggesting that there may exist other phosphorylation sites on ATF4 targeted by mTORC1 in addition to the five serine/threonine sites that we have identified. Together, these results indicate that while disruption of mTORC1-mediated ATF4 phosphorylation does not affect the basal function of ATF4 in maintaining cell growth, ATF4 phosphorylation downstream of v-ATPase/mTORC1 signaling plays a determining role in sustaining mitochondrial homeostasis and promoting survival from ROS-associated cell death in response to mitochondrial stress.

## Discussion

The activity of mTORC1 has been shown to be required for increased ATF4 expression downstream of growth signals and during mitochondrial myopathy^42,51,65^. Full inhibition of mTORC1 furthermore induces the rapid loss of ATF4 at both mRNA and protein levels^50,51^, which likely explains why previous phosphoproteomic screens for mTORC1 substrates did not manage to identify ATF4 as one of the mTORC1 phosphorylation target^66,67^, since much less ATF4 will be detected after Torin1/Rapamycin exposure. This rapid loss of ATF4 expression hence blurs the correlation of phosphorylated and nonphosphorylated ATF4 peptides before and after mTORC1 inhibition with mTORC1 activity in these two screens^50,51^. Thus, the mechanism underlying how mTORC1 regulates ATF4 function, especially during mitochondrial stress, is still not fully understood. Moreover, due to the fact that the UPR^mt^ is often considered as part of the ISR downstream of the EIF2α phosphorylation event in mammalian systems^44,45^, it has to be determined how cells distinguish stress signals from different origins, i.e., mitochondrion and ER, to accurately activate the distinct UPR^mt^ and UPR^ER^ programs, respectively.

**Fig. 7:**
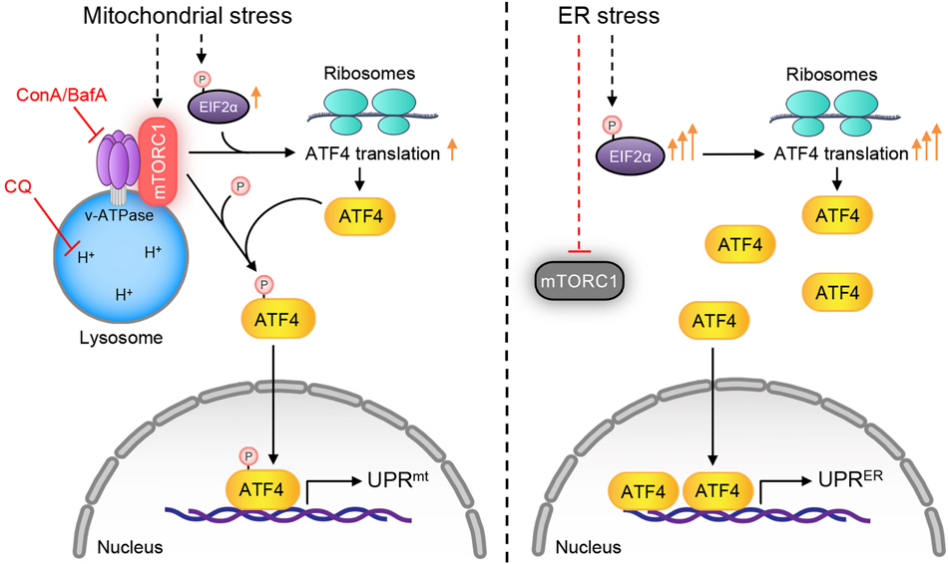
Models of how mitochondrial stress and ER stress activate mechanistically different pathways, involving the lysosomes, v-ATPase/mTORC1, ATF4, and/or ribosomes, to concordantly activate the UPR^mt^ and the UPR^ER^. Left: in response to mitochondrial stress, mTORC1 is activated at the lysosomal surface and EIF2α phosphorylation is mildly increased, leading to a moderate increase in ATF4 translation. Meanwhile, activated mTORC1 directly phosphorylates ATF4, leading to increased ATF4 binding to the promoters of UPR^mt^ genes and UPR^mt^ activation. Right: in response to ER stress, mTORC1 activity is suppressed but the EIF2α phosphorylation is robustly increased, leading to an increase in translation and nuclear accumulation of ATF4, and to the subsequent activation of the ER UPR (UPR^ER^). CQ chloroquine, ConA/BafA1 Concanamycin A/Bafilomycin A1.

Here, we reveal that mitochondrial stress and ER stress activate mechanistically different pathways, involving the lysosomes, v-ATPase/mTORC1, ATF4, and/or ribosomes, to concordantly activate the UPR^mt^ and the UPR^ER^ (Fig. 7). We found that in response to mitochondrial stress, mTORC1 is activated at the lysosomal surface via the v-ATPase, whereas EIF2α phosphorylation is only mildly increased, leading to a moderate increase in ATF4 translation (Figs. 2e and 3b). Meanwhile, activated mTORC1 directly phosphorylates ATF4, leading to increased ATF4 binding to the promoters of UPR^mt^ genes and the activation of UPR^mt^. In contrast, upon ER stress, mTORC1 activity is gradually suppressed but EIF2α phosphorylation is robustly increased, leading to a robust increase in ATF4 translation, and the subsequent activation of the UPR^ER^. Disruption of lysosomal acidification by CQ or the v-ATPase inhibitor, ConA, hence specifically suppressed the activation of the UPR^mt^ but not the UPR^ER^. In addition, in *C. elegans*, either v-ATPase or TORC1 suppression merely abrogated the UPR^mt^, but not UPR^ER^ or cytosolic UPR (UPR^CYT^)^25^. Thus, v-ATPase acts as an evolutionally conserved node relaying the stress signal specifically from mitochondria, but not ER, to the nuclear transcriptional adaptive response.

Interestingly, a dual phase of mTORC1 inhibition was found in response to classical mitochondrial inhibitors, such as antimycin and oligomycin^56^. A rapid decrease in mTORC1 activity first occurred after ~30 min inhibition of mitochondrial respiration, which is in line with an increase in AMP level and elevated AMPK activity. However, such acute AMPK-dependent mTORC1 inhibition was quickly attenulated, as evidenced by the fully restored or even boosted mTORC1 activity 1–2 h after antimycin/oligomycin^56^, or 2–4 h after Dox administration (Fig. 3a). If the presence of mitochondrial inhibition continued and energy homeostasis was not restored, a second-phase of mTORC1 inhibition was observed, as seen after 6–8 h of mitochondrial stress (Fig. 3a, b). Notably, this second-phase of mTORC1 inhibition was furthermore dependent on ATF4^56^, highlighting a key role of ATF4-mediated stress response in the reprogramming of global metabolism upon mitochondrial stress.

How exactly mitochondrial stress leads to the activation of the v-ATPase-mTORC1-ATF4 signaling remains an important direction for future work. One possibility is that the unfolded proteins/peptides accumulated during mitochondrial stress may somehow be transported from the mitochondria to the lysosomes, and get further digested to amino acids inside the lysosomes, which then activate mTORC1 via the v-ATPase^28,68,69^. Of note, mitochondrial stress, together with the unfolded mitochondrial-derived proteins/peptides, likely represents a unique intrinsic signal for mTORC1 activation by lysosome-derived amino acids^70^, which apparently differs from what is observed in response to growth factors or exogenous amino acids^70,71^. Accordingly, more mitochondrial proteins/peptides were detected in Rab5-positive endosomes after mitochondrial perturbations^72^. Moreover, a Rab5-mediated mitochondrion-endosome-lysosome pathway was activated during mitochondrial redox stress, and functions in mitochondrial quality control independent of the mitophagy process^60,73^.

Collectively, our findings identified mammalian ATF4 as a direct phosphorylation target of mTORC1, and revealed a pivotal and specific role of lysosomes and the v-ATPase/mTORC1 complex in mediating stress signal sensing and transduction from mitochondria to the nucleus in mammals. Future work will have to determine whether mTORC1-dependent ATF4 phosphorylation also contributes to the other pleiotropic functions of mTORC1 and ATF4^23,24,68,69^, under a variety of pathophysiological conditions.

## Materials and methods

### Doxycycline treatment in C57BL/6J mice

Mice were housed under a 12-h dark/12-h light cycle and were allowed ad libitum to food and water. 9–10-week-old male C57BL/6 J mice were randomly assigned to vehicle control or Doxycycline (Dox, Cat. D9891, Sigma) treatment group. The assigned mice (*n* = 5 per group) were administrated with either vehicle control or 50 mg/kg body weight of saline-dissolved Dox by intraperitoneal injection and sacrificed after 24 h of treatment. The experiment was carried out according to the institutional, Swiss national and European Union ethical guidelines and was approved by the local animal experimentation committee of the Canton de Vaud (License number, VD3478).

### RNA extraction and RNA-seq analysis

Cells or tissue powders were directly dissolved in 1 mL of the TriPure Isolation Reagent (Cat. 11667165001, Roche) and extracted using a column-based kit (Cat. 740955.250, Macherey-Nagel). RNA-seq was performed by BGI with the BGISEQ-500 platform.

For RNA-seq results, the raw data were filtered by removing adaptor sequences, contamination, and low-quality (phred quality < 20) reads. Qualified reads were then mapped to the “*Mus_musculus*.GRCm38.95” genome with STAR aligner version 2.6.0a. Reads were counted using htseq-count version 0.10.0 using these flags: -f bam -r pos -s no -m union -t exon -i gene_id. Differential expression of genes was calculated by Limma-Voom. The genes with a Benjamini–Hochberg adjusted *P* value < 0.05 were defined as statistically significant. Genes whose expressions were significantly upregulated (adjusted *P* value < 0.05) in Dox/AntiA/FCCP treatment condition; and were then down-regulated by more than 25% of the log_2_FC after ConA co-treatment, compared to the log_2_FC of Dox/AntiA/FCCP condition, were considered as v-ATPase activity-dependent. Functional clustering was conducted using the DAVID (Database for Annotation, Visualization and Integrated Discovery) database^74^. Heatmaps were generated using Morpheus (https://software.broadinstitute.org/morpheus).

### qRT-PCR

Total RNA was extracted as described above. cDNA was synthesized using the Reverse Transcription Kit (Cat. 205314, Qiagen). qRT-PCR was conducted with the LightCycler 480 SYBR Green I Master kit (Cat. 04887352001, Roche). Primers used for qRT-PCR are listed in Supplementary Table S2. Primers for mouse *Gapdh* and *Actin*, and human *GAPDH* and *ACTIN* were used as normalization controls.

### Cell culture and drug treatment

HEK293T cells (Cat. CRL-3216) were obtained from ATCC. Immortalized wild-type and *Atf4*^*−/−*^ MEFs were kindly provided by Prof. D. Ron (Cambridge Institute for Medical Research)^63^. All cell lines were validated to be free of mycoplasma contamination and maintained in Dulbecco’s modified Eagle’s medium containing 4.5 g glucose per liter and 10% fetal bovine serum. For culturing the *Atf4*^*−/−*^ MEFs, 1× non-essential amino acids (Cat. 11140050, Gibco) and 55 μM β-mercaptoethanol (Cat. 31350010, Gibco) were furthermore supplemented to the medium, as described previously^63^; wild-type MEFs were cultured at the same condition for at least one week before comparing with the *Atf4*^*−/−*^ MEFs. Plasmids expressing ATFS-1 and ATF4 were constructed by PCR amplifying from total cDNA of *C. elegans* and MEFs, respectively, and verified by sequencing. ATF4 mutants were created with the GeneArt™ Site-Directed Mutagenesis System (Cat. A13282, ThermoFisher). Plasmids expressing Myc-tagged Rheb (Plasmid #24941), Flag-tagged mTOR (Plasmid #26603) and HA-tagged Raptor (Plasmid #8513) were purchased from Addgene. Transfection was performed with the TransIT-X2 Transfection Reagent (Cat. MIR-6000, Mirus Bio). For CRISPR/Cas9-based knockdown of v-ATPase subunits, sgRNA for human *ATP6V0C* (5’-GAATAGTCGGGGCTGCTGGG-3’) and *ATP6V0D1* (5’-TCGATGACTGACACCGTCAG-3’) were cloned to lentiCRISPR v2 plasmid (Plasmid #52961, Addgene), followed by virus package and infection procedures as described previously^75^. The compounds used for treatment of cells were: Doxycycline (Cat. D9891, Sigma), Concanamycin A (Cat. C9705, Sigma), Bafilomycin A1 (Cat. S1413, Selleckchem), Cycloheximide (Cat. S7418, Selleckchem), Torin1 (Cat. S2827, Selleckchem), Rapamycin (Cat. S1039, Selleckchem), Antimycin A (Cat. A8674, Sigma), Oligomycin (Cat. 75351, Sigma), Tunicamycin (Cat. S7894, Selleckchem) and Chloroquine diphosphate (Cat. S4157, Selleckchem), with the concentrations indicated in the figure legends.

### ChIP-qPCR of MEFs

ChIP-qPCR were performed as described previously^19^. Briefly, MEFs were fixed with 1% formaldehyde for 15 min and quenched by 0.125 mM glycine. Immunoprecipitations were carried out using antibody against ATF4 (1:100, Cat. 11815, CST). Sonication was conducted for a total time of 15 min. The primers used for ChIP-qPCR are listed in Supplementary Table S2.

### Western blot assay

Proteins were extracted with Radio-immunoprecipitation Assay (RIPA) buffer supplied with protease and phosphatase inhibitors, as described previously^32^. Immunoprecipitation of Flag-tagged proteins were carried out with the anti-FLAG M2 beads (Cat. A2220, Sigma) in RIPA buffer. For western blotting, the antibodies used were: P-EIF2α (Cat. 3597, CST, 1:500), Tubulin (Cat. T5168, Sigma, 1:2000), P-S6K (Cat. 9205, CST, 1:1000), S6K (Cat. 9202, CST, 1:1000), P-S6 (Cat. 2215, CST, 1:1000), S6 (Cat. 2317, CST,1:1000), P-4E-BP1 (Cat. 9644, CST, 1:1000), 4E-BP1 (Cat. 2855, CST, 1:1000), ASNS (Santa Cruz, Cat. sc-365809, 1:1000), EIF2α (Cat. 9722, CST, 1:1000), ATF4 (Cat. 11815, CST, 1:1000), ATF5 (Cat. ab60126, Abcam, 1:1000), ATP6V0D1 (Cat. ab202899, Abcam, 1:1000), CTSB (Cat. 31718, CST, 1:1000), Vinculin (Cat. ab129002, Abcam, 1:1000), Flag-tag (F7425, Sigma, 1:1000), Myc-tag (Cat. sc-40, Santa Cruz, 1:2000), mTOR (Cat. 2972, CST, 1:1000), Phospho-S*P (Cat. 2325, CST, 1:1000; for detecting P-S166-ATF4), Phospho-ST*P (Cat. 5243, CST, 1:1000; for detecting P-T173-ATF4), and HRP-labeled anti-rabbit (Cat. 7074, CST, 1:5000), anti-rabbit (Light-Chain Specific) (Cat. 93702, CST, 1:5000, for detecting the endogenously immunoprecipitated ATF4 and its phosphorylation) and anti-mouse (Cat. 7076, CST, 1:5000) secondary antibodies.

### Imaging of mammalian cells

For Mitotracker staining of MEFs, cells were grown on glass coverslips, and MitoTracker Red CMXRos (Cat. M7512, Invitrogen) was added to the culture medium 30 min prior to imaging according to the manufacturer’s instructions. Cells were then fixed and stained with antibodies to early endosome marker Rab5 (Cat. 3547, CST, 1:200), late endosome marker Rab7 (Cat. 9367, CST, 1:200), or lysosome marker Lamp1 (Cat. 121617, Biolegend, 1:250). For imaging the lysosomal-localized mTOR, fixed MEFs were stained with mTOR (Cat. 2972, CST, 1:200), early endosome marker Rab5 (Cat. 46449, CST, 1:200), late endosome marker Rab7 (Cat. 95746, CST, 1:200), or lysosome marker Lamp1 (Cat. 121617, Biolegend, 1:250) antibody. Images were then acquired using a ZEISS LSM 700 confocal microscope and analyzed by using ImageJ with a Mito-Morphology macro^76^. For mitochondrial network analysis, at least 20 cells were analyzed for each condition. For detecting the lysosomal pH, cells were grown on 35-mm glass-bottom dishes, and were cultured to ~60% confluence. Cells were treated with 1 μM LysoSensor Green DND-189 (Cat. L7535, ThermoFisher) for 1 h, then washed twice with PBS and incubated in fresh medium for another 30 min. In the meantime, 2 μg/mL Hoechst, together with ProLong Live antifade reagent (Cat. P36975, ThermoFisher), was added into the medium for staining the nucleus before taking images.

### Mitochondrial respiration assay

OCR of cultured MEFs was determined using the Seahorse XFe96 Extracellular Flux Analyzer (Agilent Technology) according to the manufacturer’s protocol. The OCR was measured upon serial injections of 2 μM Oligomycin, 2 μM FCCP, and a mixture of 1 μM Rotenone/Antimycin A. The OCR values were normalized to total cell number. For the measurement of OCR in response to Antimycin A treatment, MEFs were treated with 2 μM Antimycin A for 24 h, and then washed three times with control medium. Cells were then let recovered in control medium for 8 h and a standard OCR measurement assay was subsequently conducted.

### Mitochondrial ROS quantification

Mitochondrial ROS levels were measured using MitoSox (Cat. M36008, Thermofisher). Cells were treated with 2 μM Antimycin A for 48 h. MEFs were then trypsinized and incubated with 2 μM MitoSox at 37 °C for 30 min. After washing twice with PBS, the cells were then analyzed with flow cytometry. Data were quantified and plotted with FlowJo software. Three independent experiments were conducted, and similar results were acquired.

### In vitro kinase assay

Kinase assays were performed as described previously^77^, with slight modifications. For kinase assay using mTORC1, HEK293T cells were transfected with plasmids expressing Flag-tagged mTOR (Plasmid #26603, Addgene) together with HA-tagged Raptor (Plasmid #8513, Addgene). 36 h post-transfection, cells were lysed in CHAPS lysis buffer (40 mM HEPES, pH 7.5, 0.3% CHAPS, 120 mM NaCl, 1 mM EDTA, 10 mM pyrophosphate, 10 mM glycerophosphate, 50 mM NaF, 1.5 mM Na_3_VO_4_ and 1× protease inhibitor Cocktail (Cat. 78430, ThermoFisher)). mTORC1 were then immunoprecitated using anti-Flag M2 Beads (Cat. A2220, Sigma). The immunoprecipitates were washed three times with the CHAPS lysis buffer and the mTOR reaction buffer (25 mM HEPES, pH 7.4, 50 mM KCl, 20% glycerol, 10 mM MgCl_2_, 4 mM MnCl_2_, 1 mM DTT), respectively. The assays were carried out in 50 μL mTOR reaction buffer with or without 200 μM ATP and/or the recombinant human His-tagged ATF4 (Cat. ab109946, Abcam) at 30 °C for 45 min. When indicated, Torin1 (250 nM, Cat. S2827, Selleckchem) was added 10 min prior to the start of the assay. Reactions were stopped by adding 4× SDS loading buffer. For kinase assays using recombinant mTOR, the recombinant GST-tagged kinase active human mTOR (Cat. PV4753, ThermoFisher) was used instead of the immunoprecipitated mTORC1. Following SDS-PAGE and SimplyBule SafeStain (Cat. LC6060, Invitrogen) staining, the bands corresponding to His-ATF4 were sliced, digested with either trypsin or GluC, and then analyzed by Liquid Chromatograph Triple Quadrupole Mass Spectrometer (LC-MS/MS) for phosphorylated peptides.

### Statistical analysis

All statistical analyses were performed using GraphPad Prism 8 software. Differences between two groups were assessed using two-tailed unpaired Student’s *t* tests. No statistical methods were used to predetermine sample size. The experiments were not randomized, and investigators were not blinded to allocation during experiments and outcome assessment. Data distribution was assumed to be normal but this was not formally tested. Analysis of variance (ANOVA) followed by Tukey post hoc test (one-way ANOVA for comparisons between groups, and two-way ANOVA for comparisons of magnitude of changes between different groups from different treatments or cell lines) was used when comparing more than two groups.

### Supplementary information

Supplementary Figures

Supplementary Table S1

Supplementary Table S2

## Data Availability

Original reagents are available upon request. The raw and processed sequencing datasets have been deposited in the NCBI Gene Expression Omnibus (GEO) database with the accession number: GSE179510.
