# Supplementary material for: Lysosomes mediate the mitochondrial UPR via mTORC1-dependent ATF4 phosphorylation
Source: Cell Discov. 2023 Sep 7;9:92. doi: 10.1038/s41421-023-00589-1 (PMC10484937; doi:10.1038/s41421-023-00589-1)
Supplement: Supplementary file 1 — Supplementary Figures [file 41421_2023_589_MOESM1_ESM.pdf]

Supplementary Fig. S1

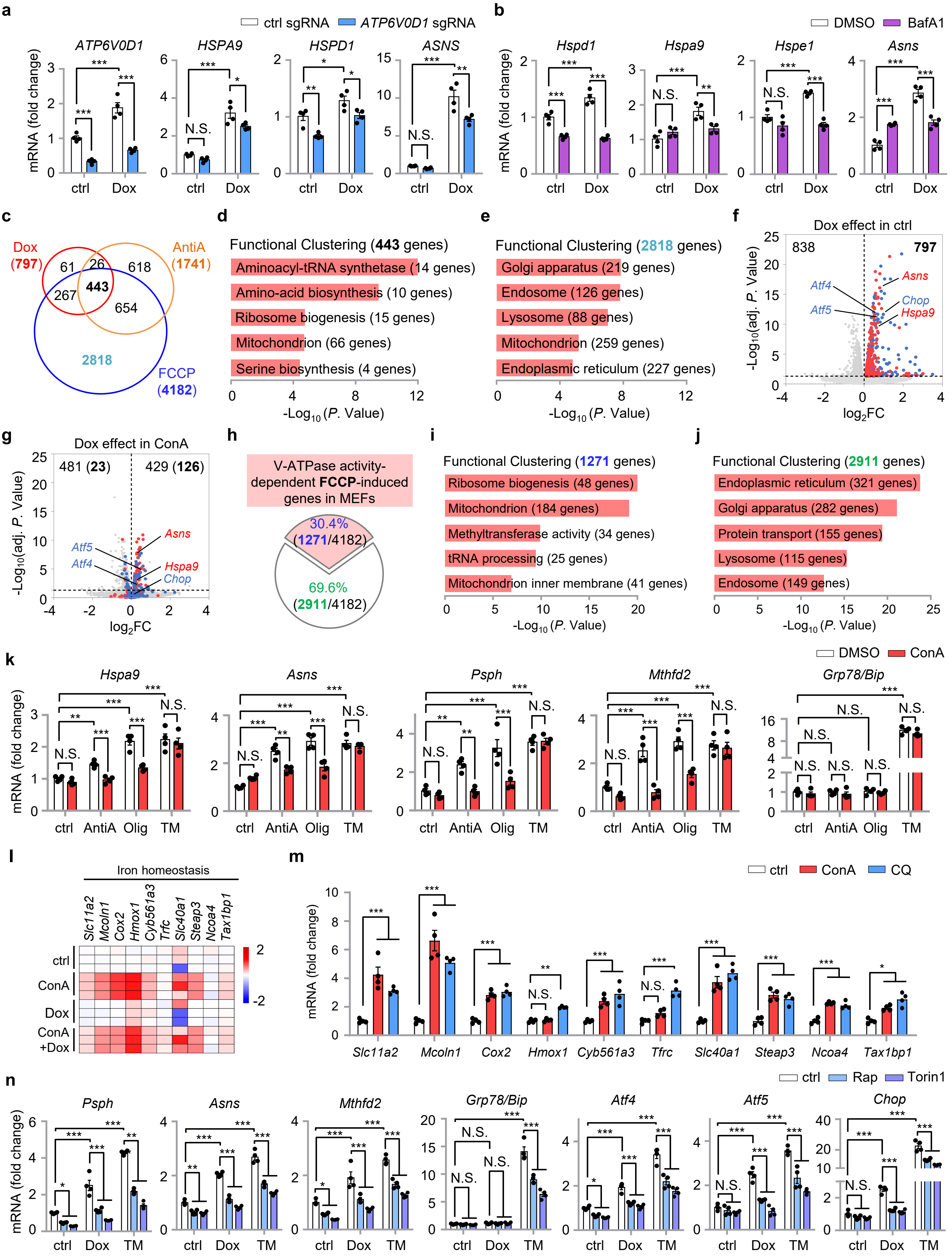

Supplementary Fig. S1 An essential role of v-ATPase and mTORC1 activity in UPR^{mt} activation. **a**, qRT-PCR results (*n* = 4 biologically independent samples) of HEK293T cells expressing control (ctrl) or *ATP6V0D1* sgRNA, and treated with or without Doxycycline (Dox) (30 µg/mL) for 24 h. **b**, qRT-PCR results (*n* = 4 biologically independent samples) of MEFs pretreated with DMSO control or Bafilomycin A1 (BafA1) (1 µM) for 1 h, and then co-treated with or without Dox (30 µg/mL) for 24 h. **c**, The up-regulated transcripts in response to Dox, Antimycin A (AntiA) or FCCP treatment based on the RNA-seq dataset. **d**,**e**, Functional clustering of the 443 (**d**) and 2,818 (**e**) genes as indicated in (**c**). **f**,**g**, Volcano plots showing the effect of Dox in control (DMSO-treated) MEFs (**f**), or in ConA-treated MEFs (**g**) (numbers in bold are the common genes within the 797 genes as indicated in (**f**)). FC, fold change. Genes whose induction upon Dox treatment is dependent on v-ATPase activity are highlighted in red, the three putative UPR^{mt} transcription factors whose induction is independent on v-ATPase activity are highlighted in blue. **h**, Diagram of the UPR^{mt} genes that are dependent (red) or independent (blue) on v-ATPase activity for induction upon FCCP treatment. **i**,**j**, Functional clustering of the 1,271 (**i**) and 2,911 (**j**) genes as indicated in (**h**). **k**, qRT-PCR results (*n* = 4 biologically independent samples) of MEFs pretreated with DMSO control or ConA (200 nM) for 1 h, and then co-treated with or without AntiA (2 µM), Oligomycin (Olig, 2 µM), or Tunicamycin (TM, 1.5 µg/mL) for 24 h. **l**, qRT-PCR results (*n* = 4 biologically independent samples) of MEFs pretreated with DMSO, Rapamycin (Rap, 100 nM) or Torin1 (250 nM) for 1 h, and then co-treated with or without Dox (30 µg/mL) or TM (1.5 µg/mL) for 24 h. **m**, Heat-map of the relative expression levels of iron homeostasis related genes in MEFs treated with Dox and/or ConA in $\log_2$ fold-change, based on the RNA-seq dataset. See Supplementary Table S1 for detailed gene expression changes. **n**, qRT-PCR results (*n* = 4 biologically independent samples) of MEFs treated with control (ctrl), 200 nM ConA or 50 µM CQ for 24 h. Error bars denote S.E.M. Statistical analysis was performed by ANOVA followed by Tukey post-hoc test (*$P < 0.05$; **$P < 0.01$; ***$P < 0.001$; N.S., not significant).

Supplementary Fig. S2

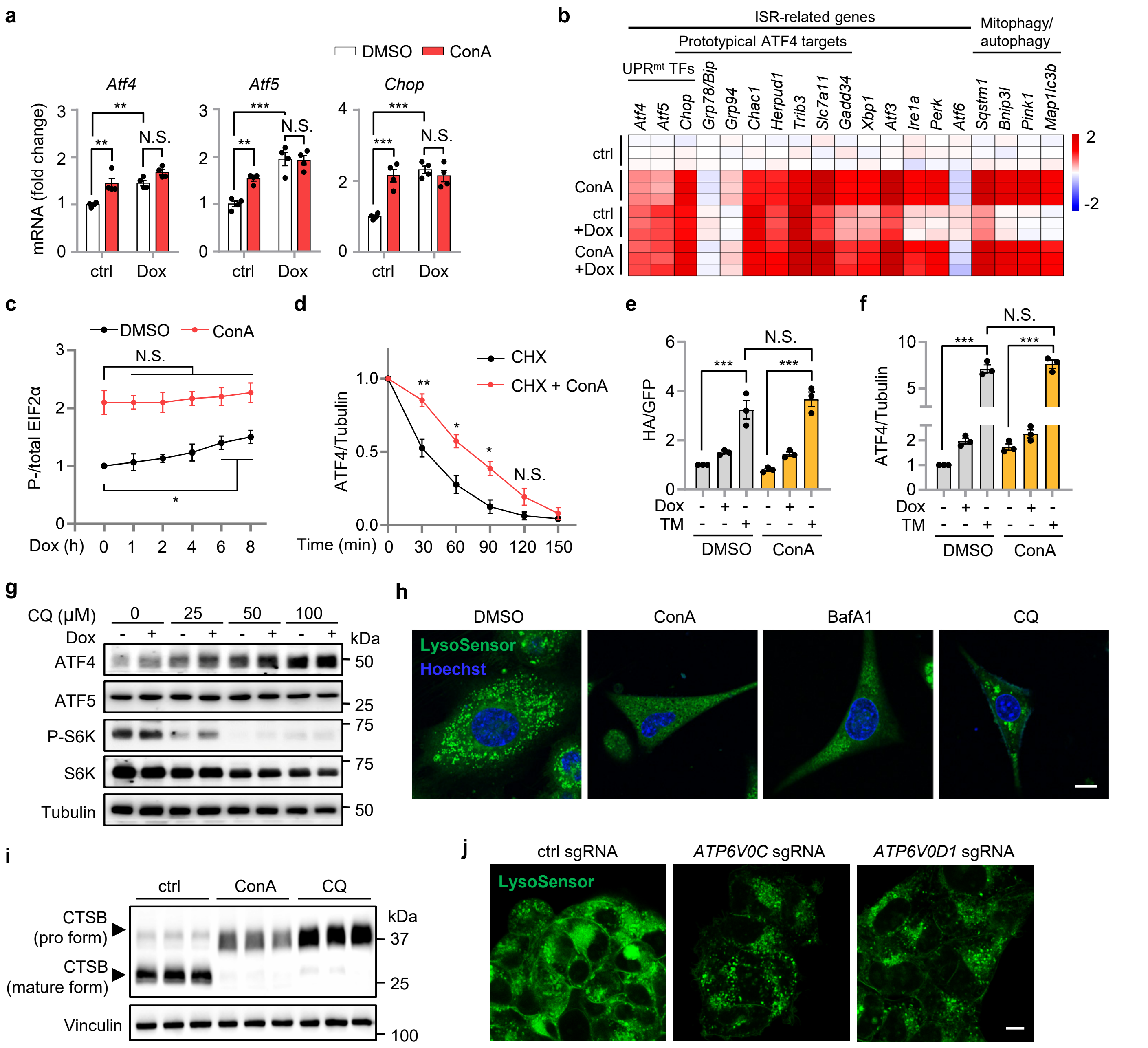

Supplementary Fig. S2 Impacts of Dox and lysosomal acidification inhibitors in gene expression and lysosomal function. a, qRT-PCR results (n = 4 biologically independent samples) of MEFs pretreated with DMSO control or v-ATPase inhibitor Concanamycin A (ConA) (200 nM) for 1 h, and then co-treated with or without Dox (30 µg/mL) for 24 h. **b**, Heat-map of the relative expression levels of ISR (integrated stress response) and mitophagy/autophagy related genes in MEFs treated with Dox and/or ConA in $\log_2$ fold-change, based on the RNA-seq dataset. See Supplementary Table S1 for detailed gene expression changes. **c**, Relative levels of EIF2α phosphorylation versus total EIF2α (n = 3 independent experiments) in conditions as shown in Fig. 2a. **d**, Relative levels of ATF4 versus tubulin (n = 3 independent experiments) in conditions as shown in Fig. 2c. **e**, **f**, Relative expression of HA-mScarlet versus GFP (**e**), and ATF4 versus tubulin (**f**) in conditions as shown in Fig. 2e. **g**, Western blots of MEFs pretreated with PBS control or 25-100 µM chloroquine (CQ) for 1 h, and then co-treated with or without Dox (30 µg/mL) for 24 h. **h**, Lysosomal acidification inhibitors disrupts lysosomal pH. Representative images of MEFs treated with for ConA (200 nM), BafA1 (1 µM) or CQ (50 µM) for 24 h, and stained for LysoSensor DND-189 (green) and Hoechst (blue). Scale bar, 10 µm. **i**, Western blots of MEFs treated with control (ctrl), ConA (200 nM), or CQ (50 µM) for 24 h. The pro and mature forms of Cathepsin B (CTSB) were as indicated. **j**, Representative images of HEK293T cells expressing control, *ATP6V0C* or *ATP6V0D1* sgRNA, and stained for LysoSensor DND-189 (green). Scale bar, 10 µm. Error bars denote S.E.M. Statistical analysis was performed by ANOVA followed by Tukey post-hoc test (*P < 0.05; **P < 0.01; ***P < 0.001; N.S., not significant).

Supplementary Fig. S3

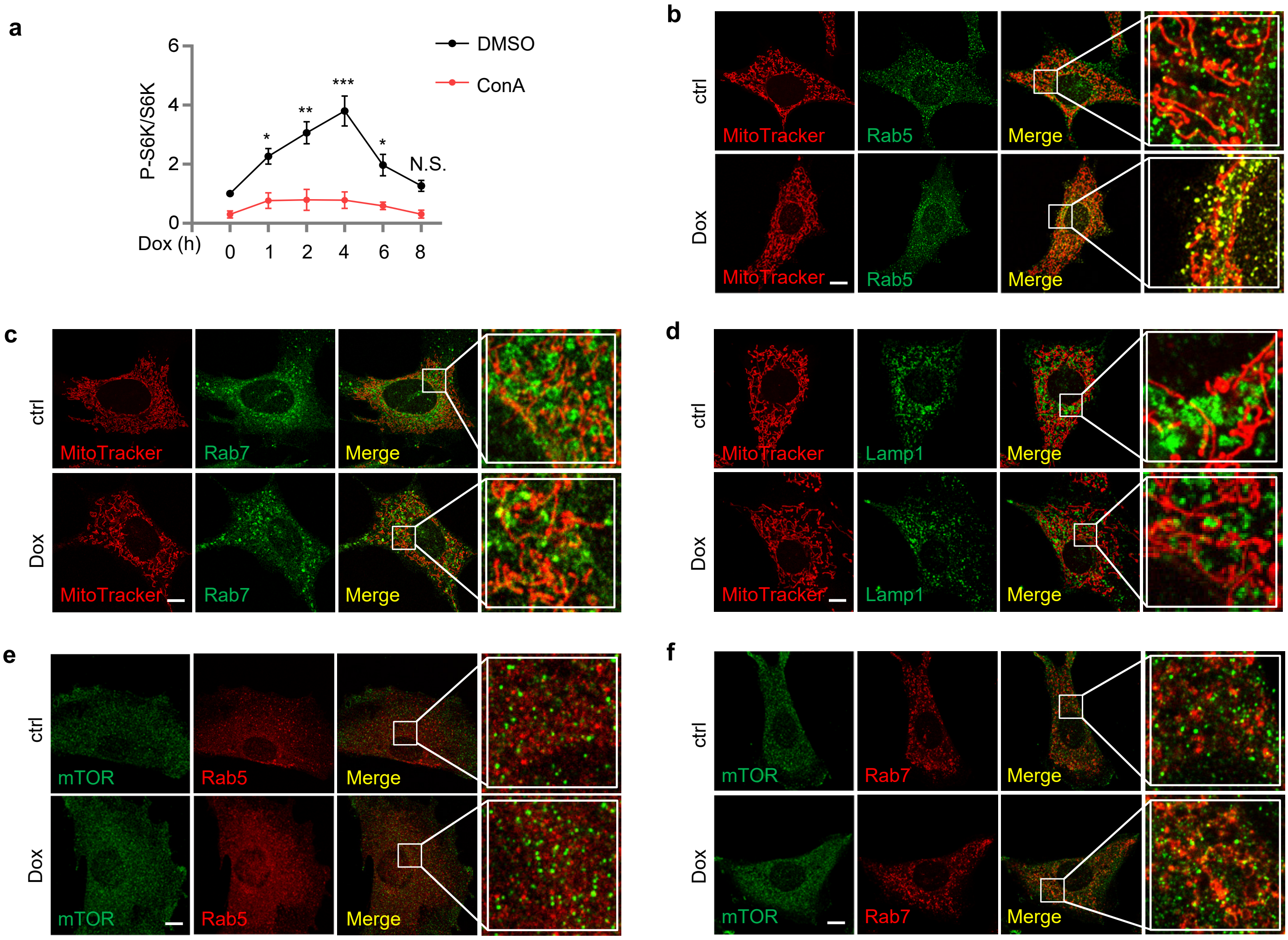

Supplementary Fig. S3 A mitochondrion-endosome-lysosome route for mTORC1 activation upon mitochondrial stress. a, Relative expression of S6K phosphorylation versus S6K total (n = 3 independent experiments) in conditions as shown in Fig. 3a. **b-d**, MitoTracker co-localizes with early endosomes (**b**), partially co-localizes with late endosomes (**c**), and does not co-localize with lysosomes (**d**), during mitochondrial stress. MEFs were stained with MitoTracker (red) for 1 h, and then treated with or without Dox (30 μg/mL) for 3 h, cells were then fixed and stained with the early endosome marker Rab5 (green) (**b**), the late endosome marker Rab7 (green) (**c**), or the lysosome marker Lamp1 (green) antibodies (**d**). **e**,**f**, mTOR does not co-localize with the early endosomes (**e**) and partially co-localizes with late endosome (**f**) during mitochondrial stress. MEFs were treated with or without Dox (30 μg/mL) for 3 h, cells were then fixed and co-stained with mTOR (green), and early endosome marker Rab5 (red) (**e**), or late endosome marker Rab7 (red) antibodies (**f**). Scale bars, 10 μm. Error bars denote S.E.M. Statistical analysis was performed by ANOVA followed by Tukey post-hoc test (*P < 0.05; **P < 0.01; ***P < 0.001; N.S., not significant).

Supplementary Fig. S4

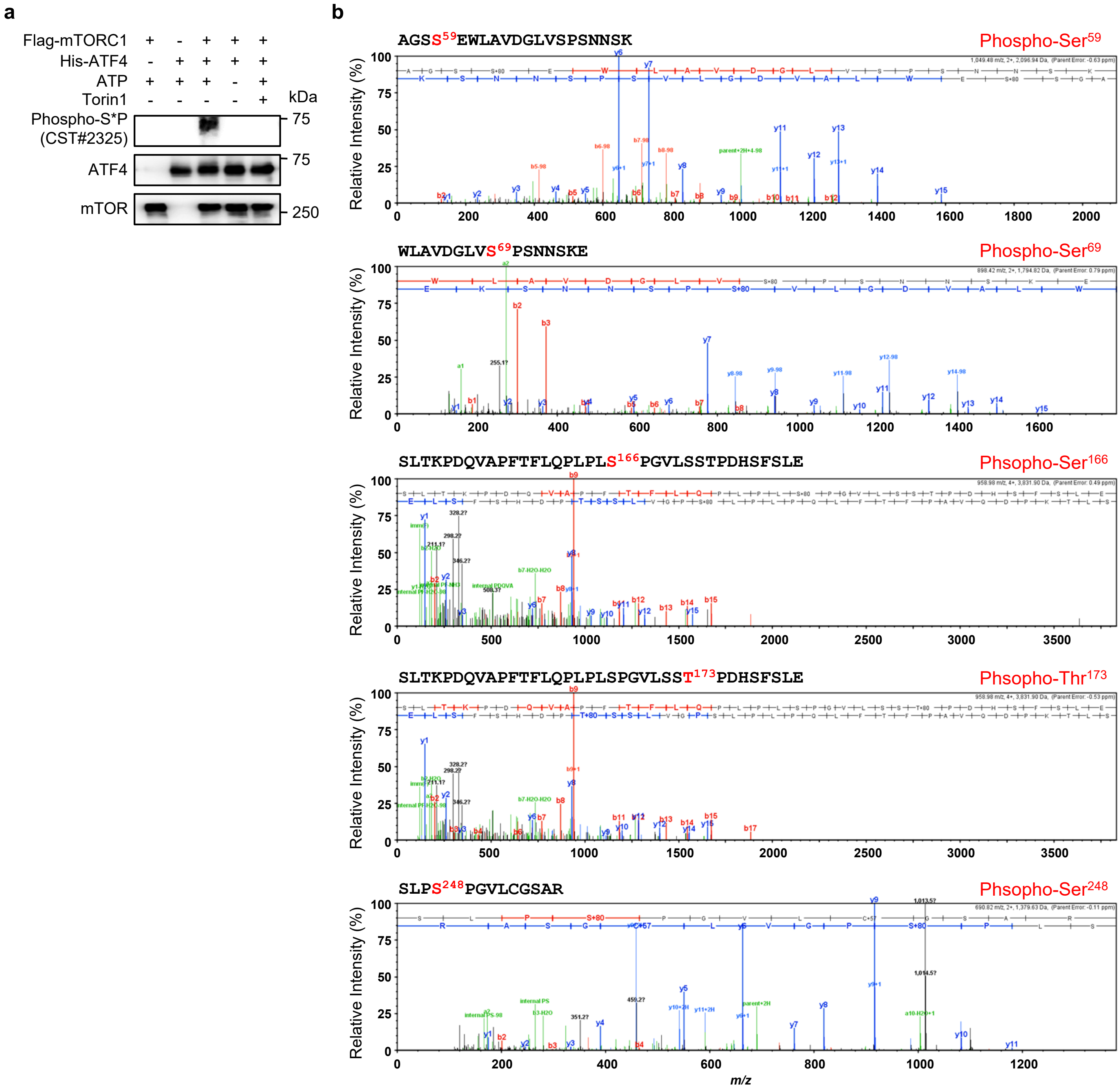

Supplementary Fig. S4 Identification of ATF4 as a direct phosphorylation substrate of mTORC1. a, mTORC1 directly phosphorylates ATF4 in vitro. In vitro kinase assay was performed with Flag-tagged mTORC1 immunoprecipitated from HEK293T cells and recombinant His-tagged ATF4, with or without Torin1 (250 nM). **b**, The representative spectrums for the phosphorylated peptides of human ATF4 identified by Liquid Chromatograph Triple Quadrupole Mass Spectrometer (LC-MS/MS), with numbering according to the amino acid sequence of human ATF4 protein.

Supplementary Fig. S5

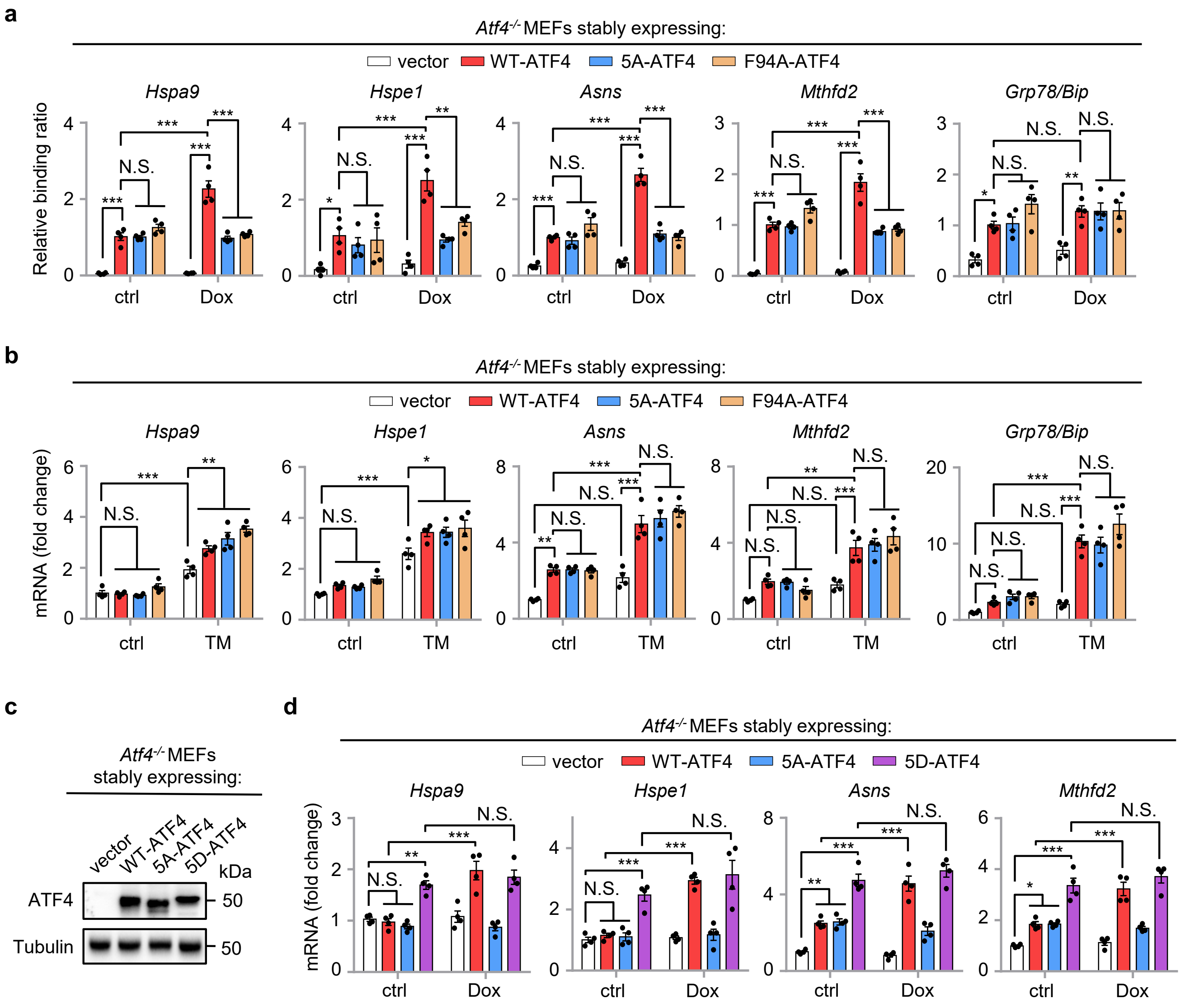

Supplementary Fig. S5 mTORC1-mediated ATF4 phosphorylation is required for mitochondrial stress-induced ATF4 binding to the promoters of UPR^{mt} genes, but non-essential for ER stress response. a, ATF4 phosphorylation defective (5A) and TOS motif disrupted (F94A) mutants failed to bind to the promoters of UPR^{mt} genes in *Atf4*$^{-/-}$ MEFs upon Dox treatment. ATF4 ChIP-qPCR analysis (n = 4 biologically independent samples) of the promoters of the UPR^{mt} genes in *Atf4*$^{-/-}$ MEFs stably expressing empty vector, wild-type, 5A or F94A forms of ATF4, with or without Dox (30 μg/mL) treatment for 3 h. **b**, qRT-PCR results (n = 4 biologically independent samples) of *Atf4*$^{-/-}$ MEFs stably expressing empty vector, wild-type, 5A or F94A forms of ATF4, with or without Tunicamycin (TM, 1.5 μg/mL) treatment for 24 h. **c**, Western blots of *Atf4*$^{-/-}$ MEFs stably expressing empty vector (vector), wild-type ATF4 (WT-ATF4), phospho-defective mutant (5A-ATF4), and the phospho-mimic mutant (5D-ATF4). **d**, qRT-PCR results (n = 4 biologically independent samples) of *Atf4*$^{-/-}$ MEFs stably expressing vector, wild-type, 5A or 5D forms of ATF4, treated with or without Dox (30 μg/mL) for 24 h. Error bars denote S.E.M. Statistical analysis was performed by ANOVA followed by Tukey post-hoc test (*P < 0.05; **P < 0.01; ***P < 0.001; N.S., not significant).

Supplementary Fig. S6

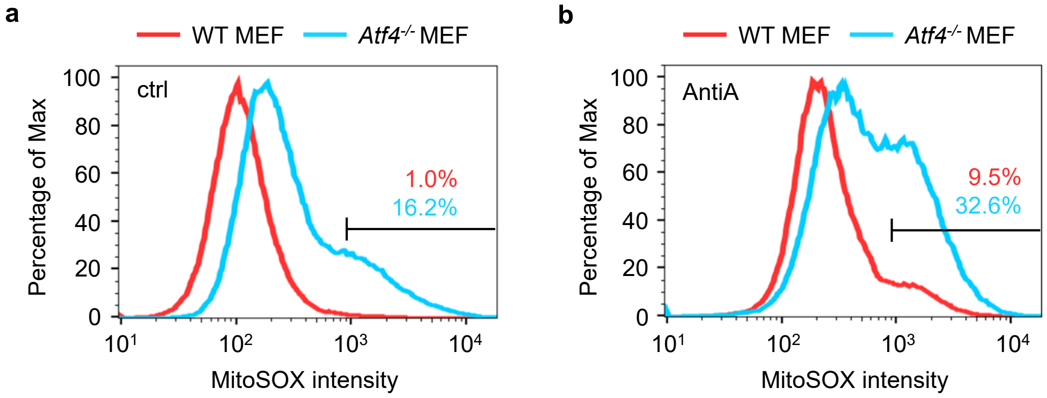

Supplementary Fig. S6 *Atf4* loss-of-function leads to disruption of mitochondrial redox homeostasis. a,**b**, Representative flow cytometry result of the mitochondrial superoxide (MitoSOX) intensity of wild-type (WT) and *Atf4*$^{-/-}$ MEFs, after DMSO control (ctrl) (**a**) or Antimycin A (AntiA, 2 µM) (**b**) treatment for 48 h. The percentages of MitoSOX-positive cells are indicated.
