# Supplementary material for: Lysosomes mediate the mitochondrial UPR via mTORC1-dependent ATF4 phosphorylation
Source: Cell Discov. 2023 Sep 7;9:92. doi: 10.1038/s41421-023-00589-1 (PMC10484937; doi:10.1038/s41421-023-00589-1)
Supplement: Supplementary file 3 — Supplementary Table S2 [file 41421_2023_589_MOESM3_ESM.pdf]

Supplementary Table S2. List of primers used for qRT-PCR and ChIP-qPCR in this study.

Species	Application	Gene	Forward primer (5'-3')	Reverse primer (5'-3')
mouse	qRT-PCR	*Hspd1*	TCTTCAGGTTGTGGCAGTCA	CCCCTCTTCTCCAAACACTG
mouse	qRT-PCR	*Hspa9*	AATGAGAGCGCTCCTTGCTG	CTGTTCCCCAGTGCCAGAAC
mouse	qRT-PCR	*Hspe1*	CTGACAGGTTCAATCTCTCCAC	AGGTGGCATTATGCTTCCAG
mouse	qRT-PCR	*Asns*	TTGACCCGCTGTTTGGAATG	CGCCTTGTGGTTGTAGATTTCAC
mouse	qRT-PCR	*Mthfd2*	CCTACAGCCCTTCCACCTG	TCCTGCTGTACTTCTTGCTTGA
mouse	qRT-PCR	*Psph*	GAGGCCGCAGTGTCTGAAAT	CACAATGCTCCGAAAGCCAC
mouse	qRT-PCR	*Cth*	ATAGTCGGCTTCGTTTCCTG	TCGGCAGCAGAGGTAACAAT
mouse	qRT-PCR	*Grp78/Bip*	ACTTGGGGACCACCTATTCCT	ATCGCCAATCAGACGCTCC
mouse	qRT-PCR	*Atf4*	GAAACCTCATGGGTTCTCCA	GAAAAGGCATCCTCCTTGC
mouse	qRT-PCR	*Atf5*	AGAGCCCCTGGCAGGTGA	CAGAGGAAGGAGAGCTGTGAAGT
mouse	qRT-PCR	*Chop*	CGGAACCTGAGGAGAGAGTG	CGTTTCCTGGGGATGAGATA
mouse	qRT-PCR	*Slc11a2*	GGGTTGGCAGTGTTTGATTG	CTGGGCTGTTAGTCATCTGG
mouse	qRT-PCR	*Cyb561a3*	GAATCGCACACCTCTACTCC	ATACATGCAGAGGTTTCAGGAG
mouse	qRT-PCR	*Mcoln1*	TGAGCCTCTTCATTGCACTC	ACGGAACTTGCCAGATGTG
mouse	qRT-PCR	*Steap3*	CCGTCCATTGCTAATTCCCTC	CGGCAGGTAGAACTTGTAGTG
mouse	qRT-PCR	*Slc40a1*	AGGAGAAAACAGGAGCAGATTAG	CCAACCGGAAATAAAACCACAG
mouse	qRT-PCR	*Ncoa4*	TCCTCAAGTATTGGGCCTTTC	GGTACATAAGGAGCCTGAAGAC
mouse	qRT-PCR	*Tax1bp1*	AAGTTTAAGGAGTGCCAGAGG	GCTGACTCCCCATTTTCTTTG
mouse	qRT-PCR	*Hmox1*	ACAGAGGAACACAAAGACCAG	GTGTCTGGGATGAGCTAGTG
mouse	qRT-PCR	*Cox2*	CTCACGAAGGAACTCAGCAC	GGATTGGAACAGCAAGGATTTG
mouse	qRT-PCR	*Tfrc*	AGTGTCAGAAAACCCAAGAGG	CGTTTCAGCCAGTTTCACAC
mouse	qRT-PCR	*Gapdh*	TGTGTCCGTCGTGGATCTGA	CCTGCTTCACCACCTTCTTGAT
mouse	qRT-PCR	*Actin*	GAGACCTTCAACACCCC	GTGGTGGTGAAGCTGTAGCC
human	qRT-PCR	*ATP6V0C*	GCTTCGTTTTTCGCCGTCAT	GTCATTCAGGGAGTTGGCGA
human	qRT-PCR	*ATP6V0D1*	CTACCTCAACCTGGTGCAGT	GTTCCTCATGTGGCGGAACT
human	qRT-PCR	*HSPA9*	TGGTGAGCGACTTGTTGGAAT	ATTGGAGGCACGGACAATTTT
human	qRT-PCR	*HSPD1*	GGGTAACCGAAGCATTTCTGC	CTGCACTCTGTCCCTCACTC
human	qRT-PCR	*ASNS*	ATCACTGTCGGGATGTACCC	TGATAAAAGGCAGCCAATCC
human	qRT-PCR	*GAPDH*	TTGGTATCGTGGAAGGACTC	ACAGTCTTCTGGGTGGCAGT
human	qRT-PCR	*ACTIN*	GTCATCACCATTGGCAATGAG	CGTCATACTCCTGCTTGCTG
mouse	ChIP-qPCR	*Hspa9*	GCTTCACGACCTCTGTCCG	AAAGACTCAAGGTCACACGGG
mouse	ChIP-qPCR	*Hspe1*	GCTCCCCTTTTCTTCCGCCT	GCTCCGGACTCTGAACTCGG
mouse	ChIP-qPCR	*Asns*	CAGAACACCTCCTGGCTCTC	AGTGACAAGACCGGTTGGAG
mouse	ChIP-qPCR	*Mthfd2*	CTGCCACTGCAGAGATGGGTG	GAGGGAAGTTGGTACCCTTGGAG
mouse	ChIP-qPCR	*Grp78/Bip*	GCTCGATACTGGCCGAGACA	CGACGACGGTTCTGGTCTG
mouse	ChIP-qPCR	*Trib3*	GGTCACAGATGGTGCAATCC	CTCTTCCGCTGCTAAAGTGC
mouse	ChIP-qPCR	*Slc7a11*	GCTGAGTAATGTTGGCGCTTTCTC	CACACCAACTTACTGGGCTGC
